# Knock-in models related to Alzheimer’s disease: synaptic transmission, plaques and the role of microglia

**DOI:** 10.1186/s13024-021-00457-0

**Published:** 2021-07-15

**Authors:** Diana P. Benitez, Shenyi Jiang, Jack Wood, Rui Wang, Chloe M. Hall, Carlijn Peerboom, Natalie Wong, Katie M. Stringer, Karina S. Vitanova, Victoria C. Smith, Dhaval Joshi, Takashi Saito, Takaomi C. Saido, John Hardy, Jörg Hanrieder, Bart De Strooper, Dervis A. Salih, Takshashila Tripathi, Frances A. Edwards, Damian M. Cummings

**Affiliations:** 1 Department of Neuroscience, Physiology and Pharmacology, University College London, Gower Street, London, WC1E 6BT UK; 2 Ludwig Maximilians Universitat, Munich, Germany; 3 Institute for Synaptic Physiology, Center for Molecular Neurobiology (ZMNH), University Medical Center Hamburg-Eppendorf, Hamburg, Germany; 4 School of Pharmacy and Biomolecular Sciences, University of Brighton, Brighton, UK; 5 Cell Biology, Neurobiology and Biophysics, Biology Department, Faculty of Science, Utrecht University, Padualaan 8, 3584 CH Utrecht, The Netherlands; 6 Department of Psychiatry and Neurochemistry, Institute of Neuroscience and Physiology, The Sahlgrenska Academy at the University of Gothenburg, Mölndal, Sweden; 7 Centre for Doctoral Training at the Institute of Health Informatics, University College London, 222 Euston Road, London, NW1 2DA UK; 8 Department of Psychology, University of Cambridge, Cambridge, UK; 9 Laboratory for Proteolytic Neuroscience, RIKEN Center for Brain Science, 2-1 Wako-shi, Saitama, 351-0198 Japan; 10 Department of Neurocognitive Science, Institute of Brain Science, Nagoya City, University Graduate School of Medical Sciences, Nagoya, Aichi Japan; 11 Dementia Research Institute, University College London, Gower Street, London, WC1E 6BT UK; 12 Reta Lila Weston Institute, UCL Queen Square Institute of Neurology, 1 Wakefield Street, London, WC1N 1PJ UK; 13 UCL Movement Disorders Centre, University College London, London, UK; 14 Institute for Advanced Study, The Hong Kong University of Science and Technology, Hong Kong, SAR China; 15 Department of Neurodegenerative Disease, UCL Queen Square Institute of Neurology, Queen Square, London, WC1N 3BG UK; 16 VIB Center for Brain & Disease Research, 3000 Leuven, KU Belgium; 17 Department of Neurosciences, Leuven Brain Institute, 3000 Leuven, Belgium; 18 Institute of Healthy Ageing, University College London, Gower Street, London, WC1E 6BT UK

**Keywords:** Synaptic transmission, Synaptic plasticity, Microglia, Alzheimer’s disease, Gene expression, Neurodegeneration, TREM2, Ageing, Amyloid beta, Plaques

## Abstract

**Background:**

Microglia are active modulators of Alzheimer’s disease but their role in relation to amyloid plaques and synaptic changes due to rising amyloid beta is unclear. We add novel findings concerning these relationships and investigate which of our previously reported results from transgenic mice can be validated in knock-in mice, in which overexpression and other artefacts of transgenic technology are avoided.

**Methods:**

*App*^NL-F^ and *App*^NL-G-F^ knock-in mice expressing humanised amyloid beta with mutations in *App* that cause familial Alzheimer’s disease were compared to wild type mice throughout life. In vitro approaches were used to understand microglial alterations at the genetic and protein levels and synaptic function and plasticity in CA1 hippocampal neurones, each in relationship to both age and stage of amyloid beta pathology. The contribution of microglia to neuronal function was further investigated by ablating microglia with CSF1R inhibitor PLX5622.

**Results:**

Both *App* knock-in lines showed increased glutamate release probability prior to detection of plaques. Consistent with results in transgenic mice, this persisted throughout life in *App*^NL-F^ mice but was not evident in *App*^NL-G-F^ with sparse plaques. Unlike transgenic mice, loss of spontaneous excitatory activity only occurred at the latest stages, while no change could be detected in spontaneous inhibitory synaptic transmission or magnitude of long-term potentiation. Also, in contrast to transgenic mice, the microglial response in both *App* knock-in lines was delayed until a moderate plaque load developed. Surviving PLX5266-depleted microglia tended to be CD68-positive. Partial microglial ablation led to aged but not young wild type animals mimicking the increased glutamate release probability in *App* knock-ins and exacerbated the *App* knock-in phenotype. Complete ablation was less effective in altering synaptic function, while neither treatment altered plaque load.

**Conclusions:**

Increased glutamate release probability is similar across knock-in and transgenic mouse models of Alzheimer’s disease, likely reflecting acute physiological effects of soluble amyloid beta. Microglia respond later to increased amyloid beta levels by proliferating and upregulating *Cd68* and *Trem2*. Partial depletion of microglia suggests that, in wild type mice, alteration of surviving phagocytic microglia, rather than microglial loss, drives age-dependent effects on glutamate release that become exacerbated in Alzheimer’s disease.

**Supplementary Information:**

The online version contains supplementary material available at 10.1186/s13024-021-00457-0.

## Background

All research models present limitations and this is particularly true for Alzheimer’s disease, where there is an urgent need to understand disease progression. Until recently, most mouse models relied on transgenesis to express genes that cause inherited forms of the disease (reviewed in [1]). This has been a useful tool to initiate rising amyloid beta (Aβ) levels, a proposed trigger for sporadic Alzheimer’s disease; however, although these mice develop substantial plaque loads, they fail to develop tau tangles or extensive neurodegeneration. Furthermore, the overexpression of *APP,* inappropriate distribution and timing of expression due to ectopic promoters and disruption of off-target genes by random transgene insertion sites have raised questions over the relevance of reported findings [2]. In particular, overexpression of *APP* results in not only raised Aβ but also other APP metabolites, the levels of which may not be increased to any substantial degree in Alzheimer’s disease [3]. Such metabolites have physiological effects (for example [4] and reviewed in [5]) that may become evident in transgenic mice but be less relevant to Alzheimer’s disease.

Transgenic mice show early alterations in synaptic transmission and plasticity (for example [6, 7–10]), alongside proliferation and activation of microglia around plaques [10–13]. These microglial changes are of particular interest, as many genes with variants associated with Alzheimer’s disease are expressed by microglia [14] and, furthermore, are also altered in transgenic mice [15–17]. These studies have led to various hypotheses concerning the physiological versus pathological roles of microglia in disease progression (discussed in [18, 19, 20]).

Given the question of relevance to human disease of phenotypes obtained in transgenic mice, we employed *App* knock-in mouse models in which the Aβ sequence within *App* has been humanised [21]. In particular we use the *App*^NL-F^ line, harbouring Swedish and Iberian/Beyreuther mutations, resulting in raised total Aβ [22] and raised Αβ42:Aβ40 ratio [23], respectively. An increase in Aβ levels and a raised Αβ42:Aβ40 ratio are also features of sporadic AD, albeit not arising from mutations in *APP* [24]. We also compare *App*^NL-F^ to *App*^NL-G-F^ mice that additionally harbour the Arctic mutation, which changes the Aβ sequence, rendering it more susceptible to fibrillation [25]. It should be noted that, similar to transgenic mice, both models display tau hyperphosphorylation but fail to develop tangles or substantial neurodegeneration, even with humanised *Mapt* (encoding the protein tau; [26]).

Here we report phenotypes consistent with and contrasting to our previously published results obtained in transgenic mice [9, 10]; initial results published in [27]. Alterations in excitatory synaptic transmission and plasticity were examined in *App* knock-in mouse hippocampus at stages prior to plaque deposition through to old age. Furthermore, we investigated the microglial contribution to these using an inhibitor of the microglial survival factor CSF1R [28], resulting in little effect on plaque development but a complex synaptic phenotype in which partial depletion of microglia had more substantial effects than near-complete ablation.

## Methods

See Additional file 1 for complete Methods.

### Animals

Experiments were performed in accordance with the UK Animal (Scientific Procedures) Act 1986 and following local ethical review. Homozygous *App* knock-in mice and wild type counterparts were group housed (2–5 mice) with ad libitum supply of food and water.

Animals were decapitated, the brain rapidly extracted and bisected. One hemisphere was drop-fixed in 10% formalin for histology; the other was processed for electrophysiology or RNA extraction.

### Genotyping

Genomic DNA was extracted and standard PCR reactions performed to identify the presence of the knock-in genes.

### Histology

Brain sections (30 μm) transverse to the long axis of the hippocampus were prepared and processed as free-floating sections and nuclei counterstained with 4′,6-diamidino-2-phenylindole (DAPI).

Plaques were labelled with luminescent conjugated oligothiophenes (LCOs; [29]).

Immunohistochemical experiments employed standard techniques [10]. Sections were permeabilised, non-specific binding blocked, then incubated with primary antibodies (1:500 rabbit anti-IBA1; or 1:500 rat anti-CD68) overnight at 4 °C, followed by Alexa-conjugated secondary antibodies for 2 h at room temperature in the dark.

### Imaging and data analysis

Epifluorescent photomicrographs of whole hippocampal regions within sections were obtained under a 20× objective by area-defined serial scanning with constant light, gain and exposure settings. Three sections per animal were assessed.

To determine cell densities, three non-overlapping areas of interest were defined and IBA1^+^ microglia counted only if the associated DAPI^+^ nucleus was evident.

To establish plaque size histograms, the hippocampal area within each section was defined and a colour intensity threshold set to differentiate LCOs signal from background and pixel coverage determined.

### Acute brain slice preparation for electrophysiology

Following decapitation, the extracted brain hemisphere was placed in ice-cold dissection artificial CSF (aCSF; raised Mg^++^ and reduced Ca^++^ ion concentrations). Brain slices (400 μm) transverse to the long axis of the hippocampus were cut, then incrementally changed through a series of heated (35 °C) aCSF solutions to a physiological recording aCSF (containing 1 mM Mg^2+^ and 2 mM Ca^2+^) bubbled with 95% O_2_/5% CO_2_.

### Patch-clamp recordings

Slices were allowed to recover at room temperature before a single slice was transferred to a submerged chamber and superfused with recording aCSF. Visualised CA1 pyramidal neurones were voltage-clamped in whole cell mode using glass electrodes (tip resistance 4–6 MΩ) filled with CsCl-based internal solution. Standard patch-clamp apparatus were used [9], with signal low-pass filtered sequentially at 10 kHz then 3 kHz and digitised at 10 kHz.

Initially, spontaneous currents were recorded in the absence of neurotransmitter receptor antagonists at a membrane holding potential of -70 mV. Around 90–95% of events under these conditions are inhibitory postsynaptic currents; the remainder being excitatory. Subsequently, excitatory postsynaptic currents were isolated by introducing gabazine. Spontaneous and miniature (with the addition of tetrodotoxin) currents were then recorded. Spontaneous and miniature currents were detected using an automated algorithm and inspected by eye for integrity.

Evoked currents were recorded in the presence of gabazine. Stimuli were applied via a patch electrode filled with aCSF and positioned in *stratum radiatum* of CA1, ~ 150–300 μm from the recording electrode. To establish failure rates, unitary responses were evoked by minimal stimulation. For all other experiments, stimulus intensity was set at near-minimal stimulation, such that ~ 50–80% of stimuli evoked a response.

### Field potential recordings in brain slices

Field potentials were recorded using standard protocols [10]. Slices were submerged in a heated (30 ± 1 °C) chamber, superfused with aCSF and allowed to recover for 1 h. Recording and stimulating electrodes (filled with aCSF, resistance ~ 2 MΩ) were both positioned in *stratum radiatum* of CA1, ~ 150–300 μm apart, to obtain dendritic excitatory postsynaptic field potentials (fEPSPs) in the absence of GABA_A_ receptor antagonists. Field potentials were low-pass filtered serially at 10 kHz and 3 kHz and digitised at 10 kHz. Stimulation intensity was set to evoke fEPSPs subthreshold to a population spike. Pairs of stimuli (50 ms inter-stimulus interval) were applied at 0·1 Hz. Long-term potentiation (LTP) conditioning consisted of three tetanus trains, each of twenty stimuli at 100 Hz, 1·5 s inter-train interval, applied at test-pulse stimulus intensity. Responses in each 1-min period were averaged and the slope of the first fEPSP and the paired-pulse ratio of each pair calculated as percent of the respective average baseline.

### Hippocampal homogenisation and RNA extraction

Whole hippocampus was extracted, snap-frozen, then homogenised in RNA lysis reagent. Chloroform was added to the lysate for phase separation. Following centrifugation, total RNA was extracted and DNA digested. Concentration and quality of RNA were assessed using a spectrophotometer and the A260/A280 ratios calculated. Total RNA solutions were stored at − 80 °C.

### Reverse transcription

RNA samples were treated with RNaseOUT and amplification grade DNaseI and placed in a thermocycler followed by an enzymatic denaturation step. Reverse-transcription was then performed using a High Capacity cDNA Reverse Transcription kit. Following a thermocycle step, cDNA was diluted and stored at − 20 °C.

### Real time-quantitative PCR

cDNA was tested in triplicate using SsoAdvanced Universal SYBR Green Supermix, both forward and reverse primers and cDNA diluted in nuclease-free water. Negative controls from the reverse-transcription protocol were included to test for the presence of genomic DNA or contamination. A blank control was included that replaced the cDNA solution with nuclease-free water. A melt curve was produced using a standard thermocycling protocol. All reactions were tested for the presence of a single PCR product.

### Microglial ablation

Microglia were removed from the brain by feeding mice the CSF1R antagonist PLX5622. Cages of male mice were randomly assigned to either control or test groups. The standard grain-based diet mice are fed was changed to a refined diet supplemented with either vehicle or PLX5622.

### Analyses

All analyses were performed by experimenters blinded to genotype and experimental conditions. Where multiple samples/cells/slices/sections were obtained from a single animal, a mean value was calculated for that animal, avoiding false replication from non-independent samples. Age- and sex-matched wild types were interleaved with *App*^NL-F^ and *App*^NL-G-F^ mice and thus act as common controls for both genotypes.

### Data availability

The data that support the findings of this study are available from the corresponding author, upon reasonable request.

## Results

### Pathology

**Fig. 1:**
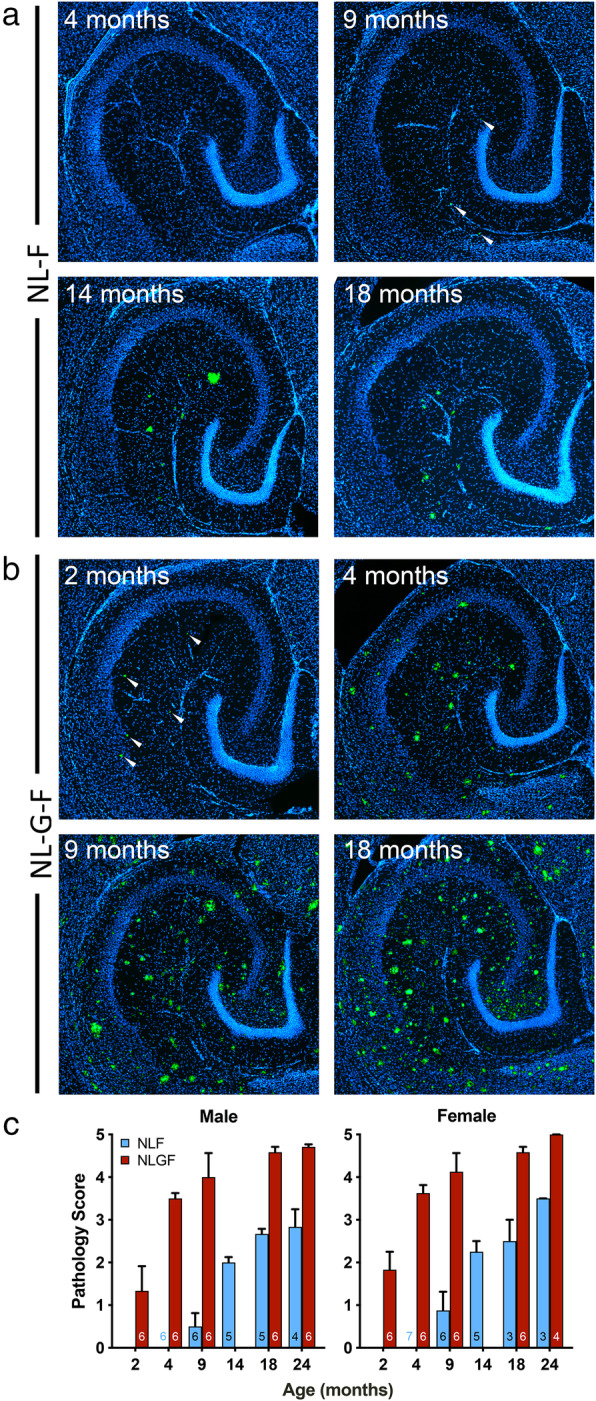
Plaques develop with age in *App*^NL-F^ and *App*^NL-G-F^ mice. **a** and **b**) Examples of plaques detected using LCOs (green) in *App*^NL-F^ (**a**) and *App*^NL-G-F^ (**b**) hippocampus at the ages indicated. DAPI nuclear counterstain (blue). **c**) Median and upper quartile range for Aβ pathology scores (0–5; Supplementary Fig.1). Sample sizes (animals) are indicated within the bars. Sections (30 μm thick) were labelled with LCOs and scored blind to experimental group. While there was a significant effect of age (*p* < 0·0001) and there were no significant differences between the sexes at any given age. (Genotype effects were not assessed by age, as the progression of plaques is known to be slower in *App*^NL-F^ than *App*^NL-G-F^.) Note that, for each animal, the score is the average of three sections, giving one value per animal which may not be an integer

Plaque development in the current *App* knock-in mice was quantified using a 0–5 rating scale of sections labelled with LCOs (Supplementary Fig. 1). In *App*^NL-F^ mice (Fig. 1a, c and Supplementary Fig. 2 for 7 months), the first sparse, small plaques were detected at 9-months-old but were not detected in all sections. By 14-months-old, plaques were reliably detected but were still small and sparse. At 18- and 24-months-old, plaques of a range of sizes were spread across the hippocampus. While pathology scores increased with age, there was no effect of sex at any given age (Kruskal-Wallis test comparing 8 groups *p* < 0·00001, followed by Dunn’s multiple comparisons between all groups, *p* > 0·5 between sexes at all ages).

In 2-months-old *App*^NL-G-F^ mice (Fig. 1b, c), only a few small plaques were detected. In 4-month-old animals, the plaque load was already heavier than in the 24-month-old *App*^NL-F^ mice. By 9-month-old, a substantial plaque load was evident, with little further increase through to the oldest ages. While there was an increase in pathology score with age, there were no significant effect of sex at any given age (Kruskal-Wallis test comparing 10 groups *p* < 0·0001, followed by Dunn’s multiple comparisons between all groups (*p* > 0·5 between sexes at all ages).

### Spontaneous and miniature excitatory postsynaptic currents

**Fig. 2:**
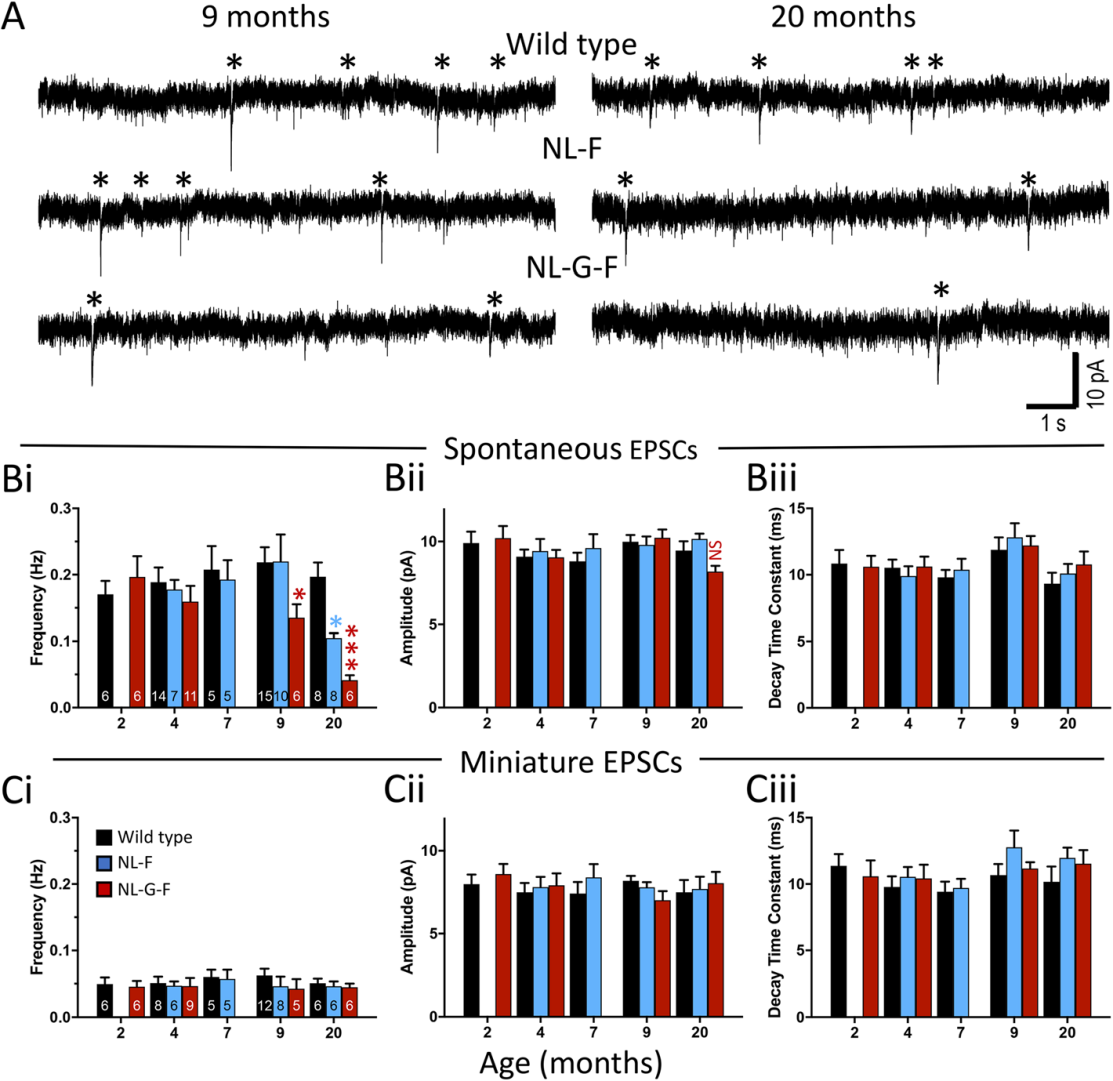
Frequency of spontaneous excitatory synaptic transmission is reduced in an age-dependent manner in *App*^NL-F^ and *App*^NL-G-F^ mice. **A**) Examples of continuous voltage-clamp recordings from CA1 pyramidal neurones in slices from 9- and 20-month-old wild type, *App*^NL-F^ and *App*^NL-G-F^ animals illustrating the reduction in frequency of spontaneous EPSCs observed at these ages. Confirmed EPSCs are indicated by the asterisks. **B**) Frequencies (i) and median amplitudes (ii) of spontaneous EPSCs in wild type and knock-in mice. **C**) Frequencies (i), median amplitudes (ii) and decay time constants (iii) of miniature EPSCs in wild type and *App* knock-in mice. Data in panels B and C plotted as mean ± SEM. Sample sizes (animals) are indicated by the numbers inside bars in subpanels i. Sequential Sidak corrected post-hoc comparisons to wild type are indicated by * *p* < 0·05; *** p < 0·001 (9-months-old: *App*^NL-G-F^ *p* = 0·025; 20-months-old: *App*^NL-F^ *p* = 0·018, *App*^NL-G-F^ *p* = 0·00028)

Synaptic transmission was examined in patch-clamp recordings of CA1 pyramidal neurones in voltage-clamp mode to record excitatory postsynaptic currents (EPSCs) in slices prepared from male mice (Fig. 2A). Recordings were made at different ages to facilitate comparisons of effects at a given stage of plaque development in the two genotypes or at the equivalent age.

The frequency of spontaneous EPSCs in slices were compared using a generalised linear mixed model (GLMM) designed to assess the effect of genotype within a given age. The genotype within age term was significant (*p* = 0·00069) and thus simple post hoc comparisons with sequential Sidak corrections were made for genotype within each age; Fig. 2Bi). There were no significant differences across the youngest ages. At 9-months-old, the lower frequency in *App*^NL-G-F^ compared to wild type reached *p* = 0·025. By 20-months-old, spontaneous EPSCs occurred at lower frequencies than wild types in both *App*^NL-F^ (*p* = 0·018) and *App*^NL-G-F^ (*p* = 0·00028) mice. There were no significant effects for EPSC amplitude or time constant (Fig. 2Bii-iii) at any age.

When miniature EPSCs were isolated, no significant differences between genotypes or ages were identified in terms of frequency, amplitude or decay (GLMM; Fig. 2 Ci-iii).

### Paired-pulse ratios

**Fig. 3:**
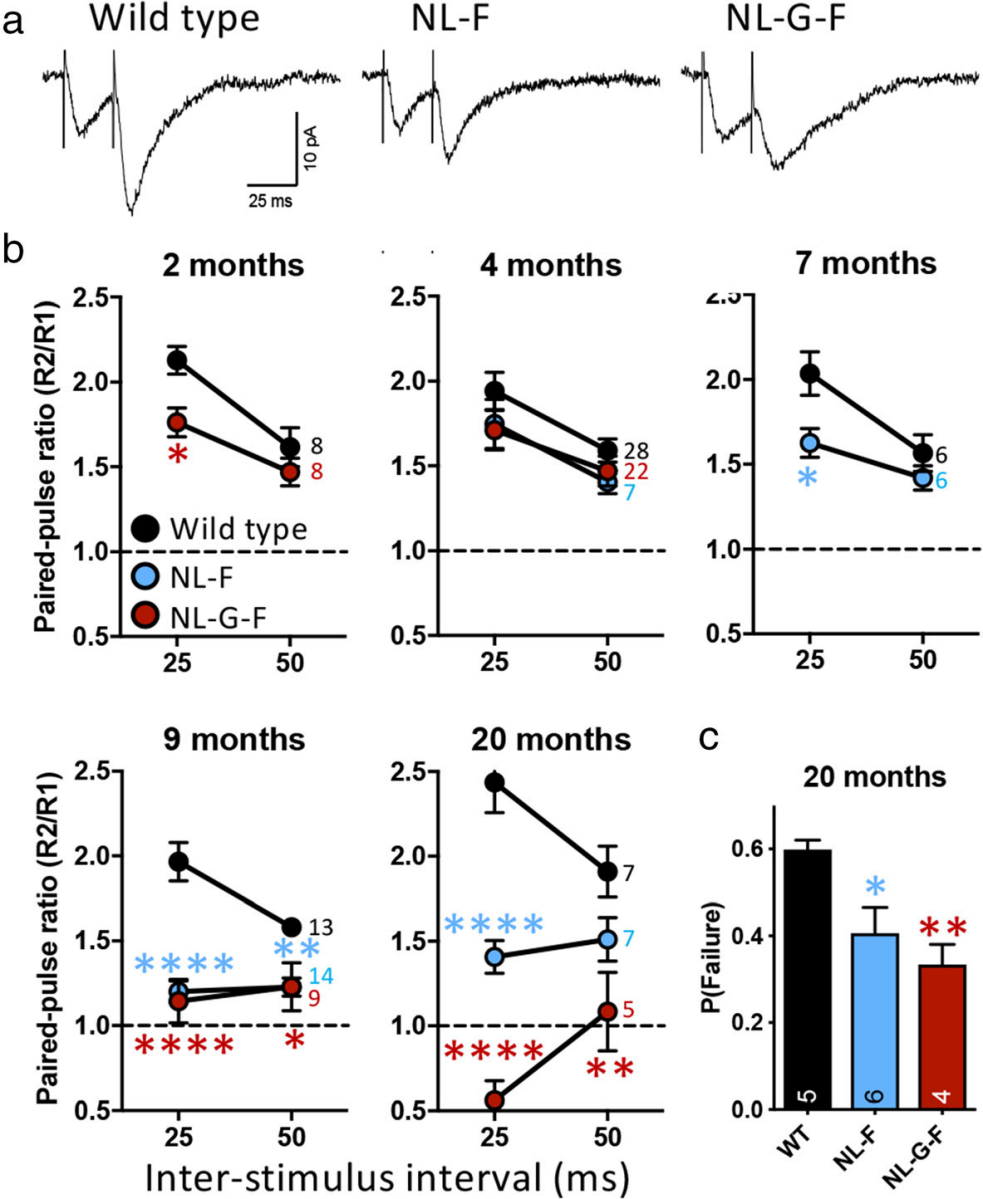
Paired-pulse ratios of evoked excitatory postsynaptic currents are altered in an age-dependent manner in *App*^NL-F^ and *App*^NL-G-F^ mice. **a**) Examples of evoked CA3-CA1 EPSCs in response to paired stimuli applied to Schaffer collaterals in slices from 20-month-old animals. **b**) Paired-pulse ratios of CA3-CA1 EPSCs at ages indicated. Sample sizes (animals) are indicated by the numbers to the right of the 50 ms data points. Holm-Sidak post hoc tests compared to wild type are indicated by * *p* < 0·05; ** *p* < 0·01; **** *p* < 0·0001 (2-month-old: *p* = 0·0174; 7-month-old: *p* = 0·019; 9-months-old *App*^NL-F^ 25 ms *p* = 3·4 × 10^−8^, *App*^NL-G-F^ 25 ms *p* = 1·1 × 10^−7^, *App*^NL-F^ 50 ms *p* = 0·0086, *App*^NL-G-F^ 50 ms *p* = 0·021; 20-month-old *App*^NL-F^ 25 ms *p* = 7.5 × 10^−5^, *App*^NL-G-F^ 25 ms *p* = 6·0 × 10^−9^, *App*^NL-G-F^ 50 ms *p* = 0·0034). **c**) Failure rates of unitary evoked EPSCs in 20-month-old wild type and *App* knock-in animals. Holm-Sidak post hoc tests compared to wild type are indicated by * *p* < 0·05; ** *p* < 0·01 (NL-F *p* = 0·013, NL-G-F *p* = 0·0069). Data in panels B and C plotted as mean ± SEM. Sample sizes (animals) are indicated by the numbers within bars

Pairs of stimuli were applied to Schaffer collaterals to evoke EPSCs (Fig. 3). Typical of CA3-CA1 synapses, paired-pulse facilitation (i.e. the second EPSC amplitude greater than the first, indicative of a low probability of glutamate release; see reference [30] for review) was observed in slices from wild type animals at all ages. As expected, facilitation decreased with longer inter-stimulus intervals.

At 2-months-old, when only rare, small plaques were detected, *App*^NL-G-F^ mice showed a reduced paired-pulse facilitation compared to wild types (two-way ANOVA, genotype × inter-stimulus interval interaction *p* = 0·0074; Sidak multiple comparisons: 25 ms *p* = 0·017; 50 ms, *p* = 0·3), indicative of a higher probability of glutamate release in *App*^NL-G-F^.

At 4-months-old, when moderate plaque deposition is seen, *App*^NL-G-F^ mice no longer showed reduced paired-pulse ratios compared to wild types. While a main effect of inter-stimulus interval remained (*p* = 0·0011), there was no effect of genotype (*p* = 0·3) nor an interaction (*p* > 0·5).

In *App*^NL-F^ mice, while there were no differences detected at 4-months-old, paired-pulse ratio was decreased at 7-months-old, before plaques were detected (main effects of genotype, *p* = 0·038 and inter-stimulus interval, p = 0·002; no interaction, p = 0·14).

Interestingly, at 9-months-old, when dense plaques were present in *App*^NL-G-F^ mice, the decreased paired-pulse ratio was again evident. *App*^NL-F^ mice also maintained the decreased paired-pulse ratio at this age. When the knock-in genotypes were compared to wild type mice, two-way ANOVA showed a significant effect of genotype (*p* = 0·0005) but no significant effect of inter-stimulus interval or an interaction. Hence, *App*^NL-F^ and *App*^NL-G-F^ mice had similarly lower paired-pulse ratios, despite the much greater plaque load in *App*^NL-G-F^ mice.

At 20-months-old, there was a significant genotype × inter-stimulus interval interaction (*p* = 0·0004). *App*^NL-F^ mice showed a similar decreased paired-pulse facilitation to that observed at 9-months-old (Sidak multiple comparisons versus wild type, 25 ms: *p* = 0·00007; 50 ms: *p* = 0·2). In *App*^NL-G-F^ mice, the effect increased further, with evoked EPSCs displaying paired-pulse depression rather than facilitation (Sidak multiple comparisons versus wild type, 25 ms *p* = 1 × 10^−8^; 50 ms *p* = 0·003).

Finally, to confirm that the lower paired-pulse ratio reflects a change in glutamate release probability, unitary responses were evoked in a subset of 20-month-old animals. The proportion of stimuli that failed to elicit a response was lower (one-way ANOVA, p = 0·008) in both *App*^NL-F^ (Holm-Sidak post-hoc test, *p* = 0·013) and *App*^NL-G-F^ (*p* = 0·007) mice compared to wild types (Fig. 3c), reflecting greater glutamate release probabilities.

### Spontaneous inhibitory postsynaptic currents

To ascertain whether there were any network-level alterations in inhibitory synaptic transmission, spontaneous currents were recorded in the absence of neurotransmitter receptor antagonists (i.e., prior to the addition of gabazine to isolate EPSCs). (It should be noted that using CsCl as an internal solution and a membrane holding potential of –70 mV gives rise to inward currents for both IPSCs and EPSCs. Subtracting the frequency of pharmacologically isolated EPSCs from this mixed population suggests that > 90% of events are inhibitory; however, isolating EPSCs using GABA_A_ receptor antagonists disinhibits the slice, increasing the frequency of EPSCs and thus IPSCs may reflect an even higher proportion in the mixed population.)

The frequency, amplitude and decay of spontaneous IPSCs (Supplementary Fig. 3a-c) were found to be not statistically different between the genotypes at any given age, suggesting that there is little net change in inhibitory synaptic transmission.

### Long-term potentiation

**Fig. 4:**
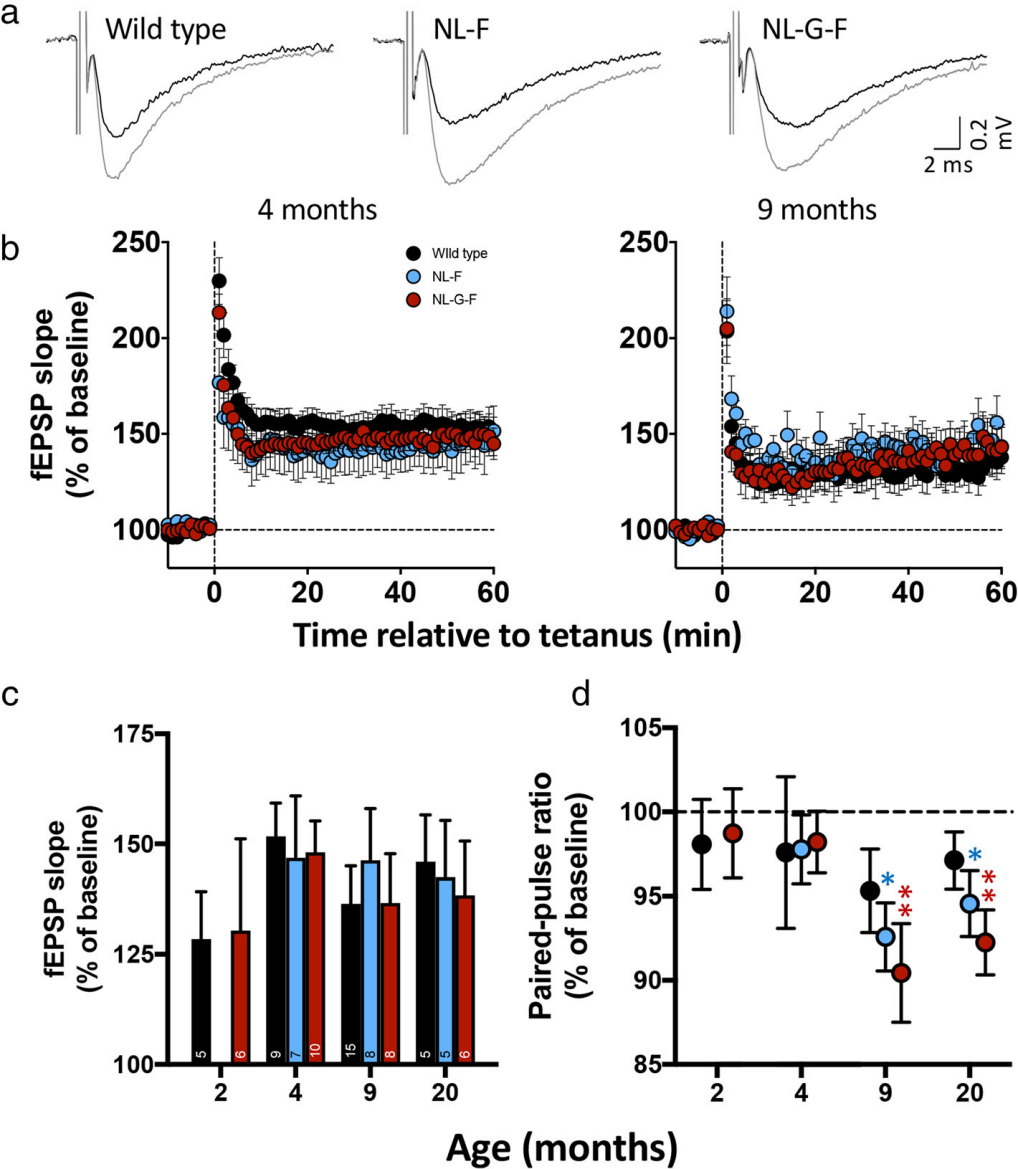
Altered locus of expression of long-term potentiation in *App*^NL-F^ and *App*^NL-G-F^ mice. **a**) Example CA3-CA1 fEPSPs recorded in *stratum radiatum* of slices from 9-month-old animals. **b**) Example time courses of field EPSP slope, expressed as *percent* of mean baseline and relative to induction tetanus. **c**) Summary of LTP magnitude across ages calculated as the mean of 51–60 min after conditioning. **d**) Paired-pulse ratio mean of 51–60 min after conditioning as a percentage of baseline. Data in panels C and D plotted as mean ± SEM. Sample sizes (animals) are indicated in the bars within panel C. NB, at 9 and 20 months, while the paired-pulse ratio was significantly different from baseline for both *App* knock-in lines and not for wild type, there was no significant difference between the groups (One sample t-tests versus 100%: * *p* < 0·05; ** *p* ≤ 0·01; 9-months-old *App*^NL-F^ *p* = 0·0080, *App*^NL-G-F^ *p* = 0·014; 20-months-old *App*^NL-F^ *p* = 0·049, *App*^NL-G-F^ *p* = 0·010)

Dendritic fEPSPs were recorded from CA3-CA1 synapses (Fig. 4). The magnitude of LTP, determined as the mean percent of baseline at 51–60 min after a moderate tetanic conditioning stimulus, was similar between genotypes across all ages (Fig. 4c).

Although the magnitude of LTP was not affected in either *App*^NL-F^ or *App*^NL-G-F^ mice, the older mice displayed a possible change in the locus of expression (i.e. presynaptic versus postsynaptic alterations underlying the expression of LTP; see [31] for review). In wild types, although paired-pulse facilitation tended to decrease after induction of LTP compared to baseline (Fig. 4d), this was not statistically significant (one sample t-test, 9-months-old *p* = 0·085; 20-months-old *p* = 0·16), suggesting a largely postsynaptic locus of expression. In contrast, paired-pulse facilitation in *App*^NL-F^ decreased compared to baseline following LTP induction (9-months-old *p* = 0·0080; 20-months-old *p* = 0·049) and the significance of this change following LTP induction was higher in *App*^NL-G-F^ (9-months-old *p* = 0·014; 20-months-old *p* = 0·010). This change in paired-pulse ratio within genotypes suggests a significant contribution of presynaptic mechanisms to LTP expression; however, there was no significant difference across genotypes at either age.

### Microglial immunohistochemistry

**Fig. 5:**
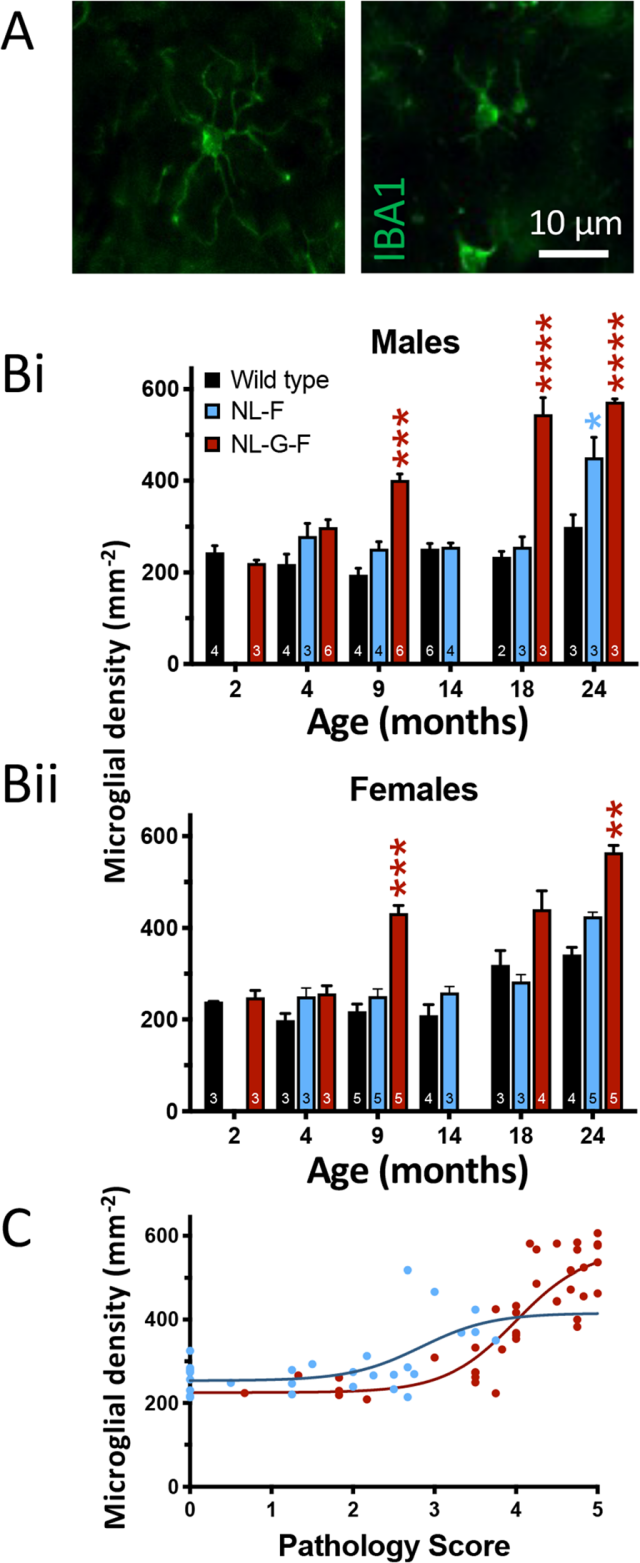
Microgliosis occurs at an earlier plaque pathology stage in *App*^NL-F^ than *App*^NL-G-F^ mice. **A**) Example of a ramified (*left*) and amoeboid (*right*) microglial cell in CA1 of 9-month-old *App*^NL-G-F^ mice. Scale bar for both images shown on right image. **B**) Densities of IBA1^+^ microglia in male (panel *i*) and female (panel *ii)*. A generalised linear mixed model, with comparisons of Age × Genotype within Sex was followed by a sequential Sidak post hoc analysis. ** *p* < 0.01; *** *p* < 0.001; **** *p* < 0.0001 with respect to age- and sex-matched wild type (Males: 9-month-old *p* = 0·00048; 18-month-old *p* = 2·2 × 10^−6^; 24-month-old *App*^NL-F^ *p* = 0·016, *App*^NL-G-F^ F *p* = 1·4 × 10^−5^; Females; 9-month-old *p* = 0·00031; 24-month-old *p* = 0·0086). Data plotted as mean ± SEM. Sample sizes (animals) are indicated within the bars. **C**) Sigmoidal fit to microglial density plotted against corresponding pathology score for an individual animal. Genotype is indicated by colour (red: *App*^NL-G-F^; blue: *App*^NL-F^); both male and female data are included

To determine the microglial response in *App* knock-in mice, we performed immunohistochemistry for IBA1 (encoded by *Aif1*) and counted CA1 microglia (Fig. 5A). The data were analysed using a GLMM permitting assessment of age × genotype interactions within either male or female mice.

Both age (*p* = 2·1 × 10^−15^) and genotype (*p* = 0·0010) had main effects on microglial density, while there was no significant effect of sex (*p* = 0·9). Moreover, there was a significant age × genotype interaction within sex (*p* = 0·000021). Sequential Sidak comparisons indicated that microglial density was greater in *App*^NL-F^ than age- and sex-matched 24-months-old wild types (Fig. 5B); and that microglial densities in *App*^NL-G-F^ were greater than age- and sex-matched wild types from 9- to 24-months-old.

To assess the effect of plaque load on microglial density, microglial density for each mouse was plotted against its corresponding pathology score (Fig. 5C). Fitting sigmoidal curves differentiated the *App*^NL-F^ and *App*^NL-G-F^ data (*p* = 0·0008). The sigmoidal fits (*App*^NL-F^ R^2^ = 0·52; *App*^NL-G-F^ R^2^ = 0·76) indicated that *App*^NL-F^ develop microgliosis with a lower plaque load than the *App*^NL-G-F^.

### Microglial gene expression

**Fig. 6:**
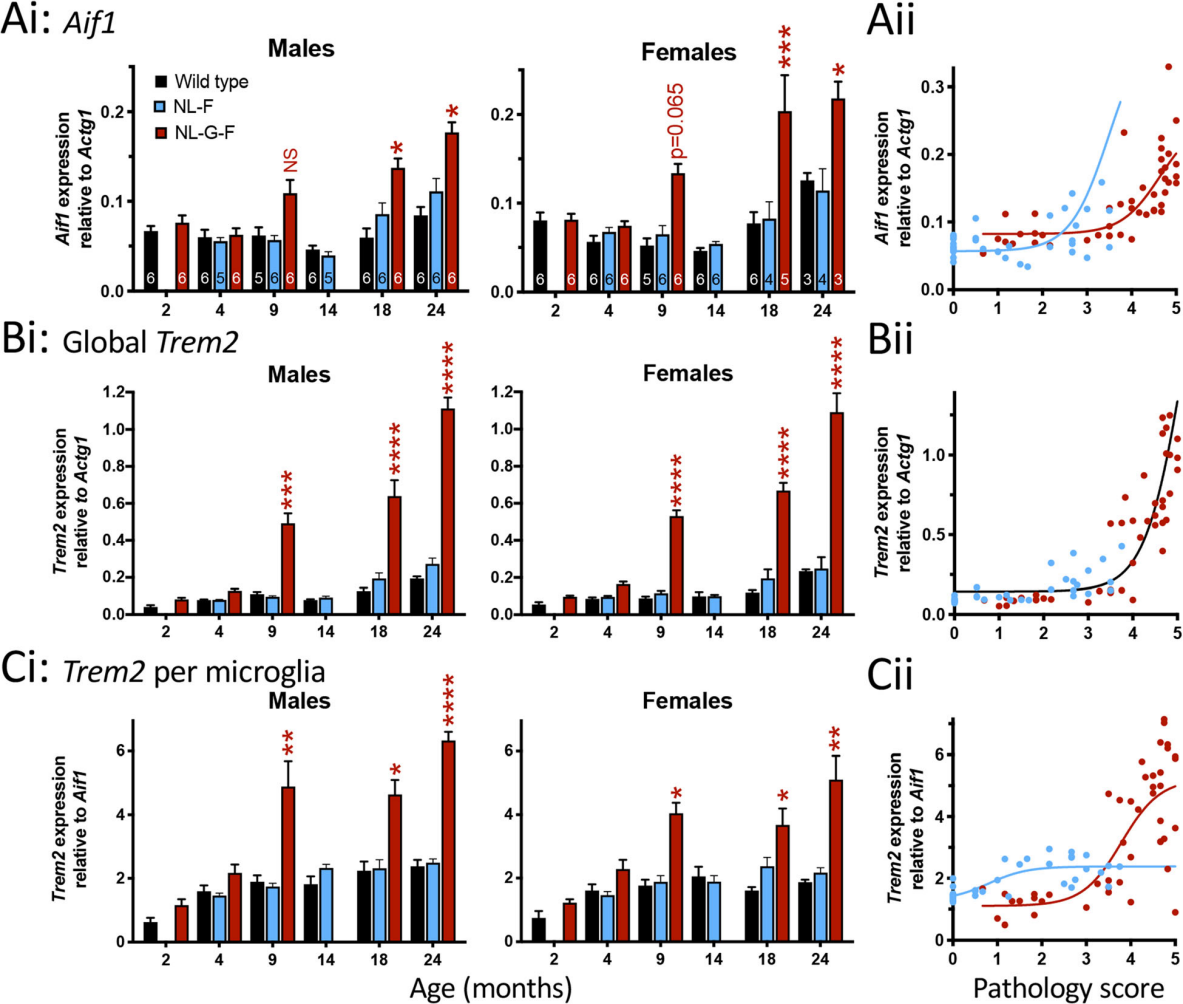
Microglial gene expression is upregulated at an earlier plaque pathology stage in *App*^NL-F^ than *App*^NL-G-F^ mice. Ai) *Aif1* (the gene encoding IBA1) gene expression determined by RT-qPCR expressed relative to the housekeeping gene *Actg1* for males (*left*) and females (*right*). Sequential Sidak post hoc analyses following GLMM are indicated by * *p* < 0·05; ** p < 0·01; *** *p* < 0·001; **** *p* < 0·0001; NS not significant (Males: 9-month-old *p* = 0·36, 24-month-old *p* = 0·026; Females: 18-months-old *p* = 0·0013, 24-month-old *p* = 0·033). Aii) Sigmoidal fit to *Aif1* expression plotted against corresponding pathology score for an individual animal. Bi) *Trem2* gene expression relative to the housekeeping gene *Actg1*, representing a global *Trem2* expression in the hippocampus for males (*left*) and females (*right*). Sequential Sidak post hoc analyses following GLMM are indicated by *** *p* < 0·001; **** *p* < 0·0001 (Males: 9-months-old *p* = 0·00015, 18-months-old *p* = 4·0 × 10^−7^, 24-month-old *p* < 1 × 10^−20^; Females: 9-months-old *p* = 1·5 × 10^−5^, 18-months-old *p* = 8·2 × 10^−8^, 24-months-old *p* = 6·7 × 10^−14^). Bii) Sigmoidal fit to *Trem2* expression relative to *Actg1* plotted against corresponding pathology score for an individual animal. Ci) *Trem2* gene expression relative to *Aif1* gene expression, representing a per-microglia *Trem2* expression for males (*left*) and females (*right*). Sequential Sidak post hoc analyses following GLMM are indicated by *p < 0.05; ** *p* < 0·01; **** *p* < 0·0001 (Males: 9-months-old *p* = 0·0020, 18-months-old *p* = 0·019, 24-months-old *p* = 1·1 × 10^−5^; Females: 9-months-old *p* = 0·026, 18-months-old *p* = 0·042, 24-months-old *p* = 0·0025). Cii) Sigmoidal fit to *Trem2* expression relative to *Aif1* plotted against corresponding pathology score for an individual animal. Sequential Sidak post hoc analyses following GLMM are indicated by * *p* < 0·05; ** *p* < 0·01; *** *p* < 0·001; **** *p* < 0·0001; NS not significant (*p* = 0·36). Data in subpanels i within A-C plotted as mean ± SEM. Sample sizes (animals) are indicated within the bars in panel A

To complement the immunohistochemical analysis of microgliosis, we used RT-qPCR to determine *Aif1* levels in whole ipsilateral hippocampus of the same mice used for immunohistochemistry. Similar to the immunohistochemical data, *Aif1* gene expression increased with age (GLMM; *p* = 4·7 × 10^−7^). There was a main effect of genotype (*p* = 0·053) and sex (*p* = 0·0037). The age × genotype interaction within sex reached *p* = 0·05 and thus sequential Sidak comparisons were made. In both sexes, *Aif1* expression tended to be higher in *App*^NL-G-F^ than wild types from 9-months-old. In females, this reached significance at 18- and 24-months-old, while in males significance was only obtained at 24-months-old (Fig. 6Ai).

Fitting sigmoidal curves to the within animal comparison of *Aif1* expression to Aβ pathology score differentiated between *App*^NL-F^ and *App*^NL-G-F^ (Fig. 6Aii; *p* < 0.0001; *App*^NL-F^: R^2^ = 0.2; *App*^NL-G-F^: R^2^ = 0.4). Thus, *Aif1* expression increases at earlier plaque stages in *App*^NL-F^ than in *App*^NL-G-F^ mice.

### *Increased expression of the microglial gene* Trem2

For further characterisation of the microglial response, the gene expression levels of the Alzheimer’s disease-associated microglial gene *Trem2* were examined (Fig. 6B). Initially, *Trem2* levels were normalised to *Actg1* (encoding gamma-actin), the same house keeping gene used for the *Aif1* experiment above. These values reflect global hippocampal *Trem2* expression. The GLMM described above was employed, revealing significant effects of age (*p* < 1 × 10^−20^) and genotype (*p* = 4·0 × 10^−9^) but not sex (*p* = 0·3). The age x genotype within sex interaction was also highly significant (*p* = 5·5 × 10^−13^). Sequential Sidak post hoc tests revealed that, for both sexes, there was a significant increase in *Trem2* expression from 9-months-old in *App*^NL-G-F^ but not *App*^NL-F^ mice (Fig. 6Bi).

To confirm that the increase in *Trem2* expression was not a simple reflection of microglial proliferation (Fig. 5), the expression of *Trem2* was subsequently normalised to *Aif1* levels to give an estimate of *Trem2* expression independent of microglial numbers. The effects of age (GLMM; *p* = 5.8 × 10^−10^) and genotype (*p* = 0.0023) were maintained and an effect of sex became apparent (*p* = 0.012). The age × genotype within sex interaction term also remained significant (*p* = 0.021). Sequential Sidak comparisons indicated significant increases in *Trem2* expression per microglial cell in *App*^NL-G-F^ mice from 9-months-old but no changes in *App*^NL-F^ mice (Fig. 6Ci).

Comparisons of *Trem2* expression to Aβ pathology scores between *App*^NL-F^ and *App*^NL-G-F^ mice were made. When normalised to *Actg1* (Fig. 6Bii), sigmoidal fits to the *Trem2* expression relationship to Aβ pathology score were unable to distinguish between the *App*^NL-F^ and *App*^NL-G-F^ mice (*p* = 0·7) and thus a single curve was used to fit both the *App*^NL-F^ and *App*^NL-G-F^ data sets (R^2^ = 0·72). In contrast, when *Trem2* expression was normalised to *Aif1* expression (Fig. 6Cii), the relationship to Aβ pathology was fit by separate sigmoidal curves (*p* = 0·037; *App*^NL-F^ R^2^ = 0·1; *App*^NL-G-F^ R^2^ = 0·56). Thus, *Trem2* expression per microglia increases at earlier Aβ plaque pathology stages in the *App*^NL-F^ mice than in the *App*^NL-G-F^ but the maximal level of expression observed in *App*^NL-F^ is less than that in the *App*^NL-G-F^ mice.

### Microglial ablation – plaques and microglial activation

**Fig. 7:**
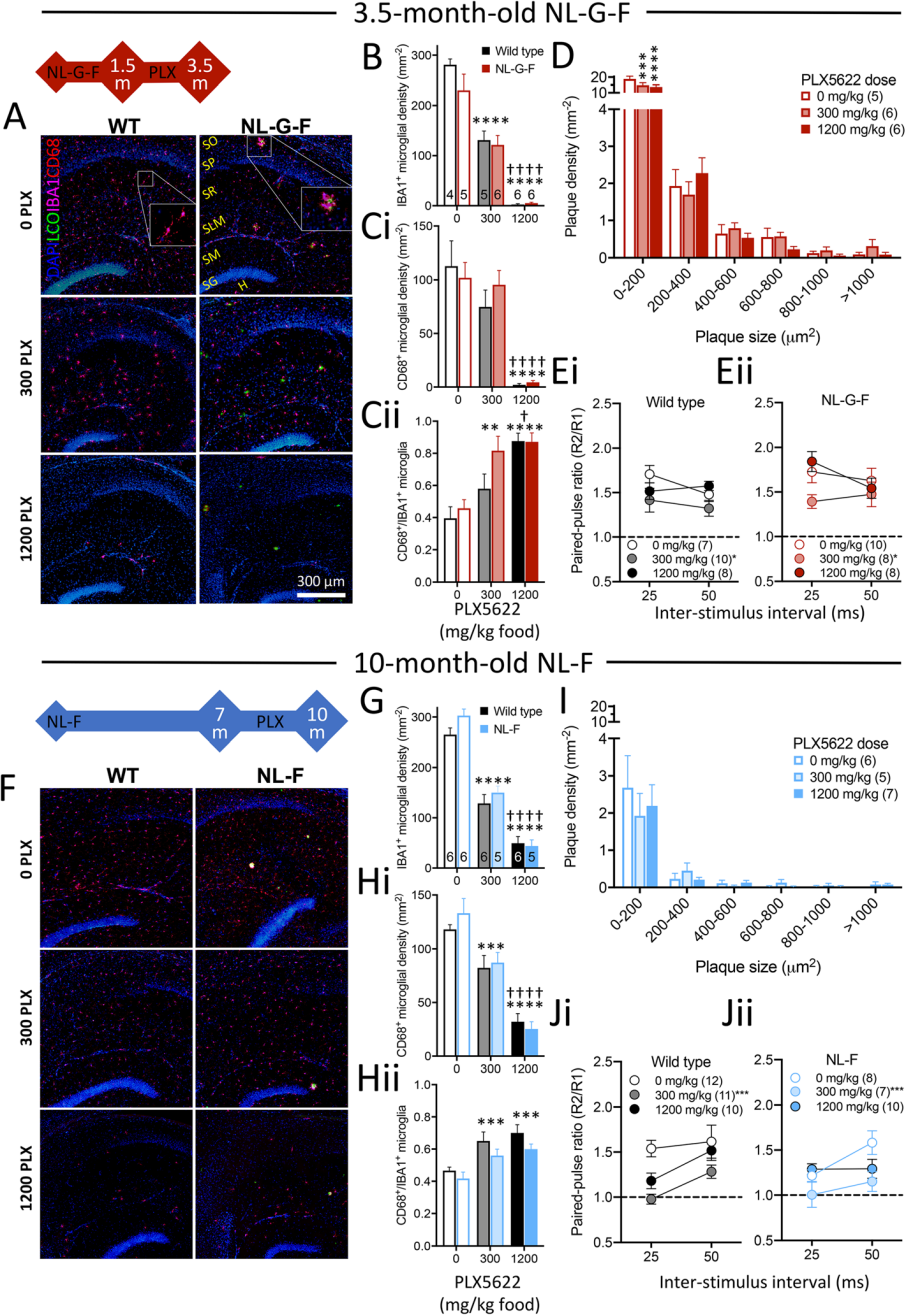
Depletion of microglia using PLX5622 reduces the presence of small plaques but exacerbates the paired-pulse ratio effects of *App* knock-in mice. *Red and blue timelines* indicate the PLX5622 feeding regimen for *App* knock-in mice and age-matched wild types. The first age indicates the start of treatment, the second the age animals were killed for experimentation. A&F) Fluorescent micrographs of *App*^NL-G-F^ (A) and *App*^NL-F^ (F) CA1 region of the hippocampus following labelling of Aβ with LCOs and fluorescent immunohistological staining for IBA1 and CD68. *Stratum oriens* (SO), *stratum pyramidale* (SP), *stratum radiatum* (SR) and *stratum lacunosum moleculare* (SLM) are indicated in the no-drug *App*^NL-G-F^ condition (for further orientation, the *stratum moleculare* (SM), *stratum granulosum* (SG) and hilus (H) within the dentate gyrus are also shown). Scale bar in NL-G-F 1200 mg/kg PLX image is 200 μm. B&G) Densities determined as cells that were IBA1^+^ microglia. Two-way ANOVA revealed significant main effects of drug (*App*^NL-G-F^ *p* < 0·0001; *App*^NL-F^ *p* < 0·0001). C&H) Densities of IBA1^+^ microglia that were also CD68^+^ in the CA1 region of *App*^NL-G-F^ (C) or *App*^NL-F^ mice. Data are presented as total density (i) and proportion of IBA1^+^ microglia (ii). Sample sizes (animals) for panels B and C are indicated within the bars of panel B; and for panels G and H are indicated in panel G. In panels B,C,G&H, Sidak corrected simple comparisons within drug are indicated by * versus control (0 mg PLX5622/kg food) and † versus 300 mg/kg; † *p* < 0·05; ** *p* < 0·01; *** *p* < 0·001; ****/†††† *p* < 0·0001. D&I) Plaque size histograms. Dunnett’s post hoc test for within plaque size bin comparisons to 0 mg/kg PLX5622 are indicated by *** p < 0·001 and **** p < 0·0001. Sample sizes (animals) are indicated within the parentheses in the legends. E&J) Paired-pulse ratios from PLX5622-treated. In wild type (Ei and Ji) and *App*^NL-G-F^ (Eii) or *App*^NL-F^ (Jii) mice. Sample sizes are indicated in the legend. For simplicity in panels E&J, only Sequential Sidak comparisons within drug dose versus control at the 25 ms inter-stimulus interval are indicated * *p* < 0·05; **** *p* < 0·0001; see main text for further details. Data in panels B-E and G-J plotted as mean ± SEM

To understand the effect of microglia on plaque formation and subsequently explore interactions with synaptic transmission we inhibited the microglial survival factor CSF1R using PLX5622 [28]. Male mice were fed starting from ages prior to plaque detection (1·5-months-old in *App*^NL-G-F^ and 7-months-old in *App*^NL-F^, along with age-matched wild type controls) and continuing until plaques were reliably detected. Following 2 (*App*^NL-G-F^; Fig. 7A&B) or 3 (*App*^NL-F^; Fig. 7F&G) months of treatment, microglial density was partially (300 mg PLX5622/kg food) or almost completely (1200 mg/kg) ablated, irrespective of genotype and age. Two-way ANOVAs within each age group revealed main effects of drug (*p* < 0·0001) but no effects of genotype (*p* = 0·2) or an interaction (*p* = 0·3). Sidak corrected simple comparisons within drug revealed significance between all doses within each age group (Fig. 7F&G).

Irrespective of genotype, PLX5622 decreased densities of microglia that were CD68^+^, a marker for a phagocytic activation state (Fig. 7Ci&Hi). Two-way ANOVA revealed a significant main effect of drug (p < 0·0001 at both ages) but no effect of genotype (*p* > 0·5 at both ages) nor an interaction (*p* > 0·5 at both ages). Sidak corrected simple comparisons within drug indicated that all doses were significantly different from each other (Fig.7Ci&Hi) except for 300 mg/kg versus 0 mg/kg within the 3·5-month-old group (*p* = 0·3).

Notably, although the number of CD68^+^ microglia decreased, the proportion of the remaining microglia that were CD68^+^ increased with drug treatment (Fig. 7Cii&Hii; two-way ANOVA, *p* < 0.0001 for both age groups). There were no significant main effects of genotype for either age group (*p* = 0·11 and *p* = 0·078, respectively), nor were there drug × genotype interactions (*p* = 0·2 and *p* = 0.8, respectively). Sidak corrected simple comparisons within drug indicated that all doses were significantly different from each other (Fig. 7Cii&Hii) except for 1200 mg/kg versus 300 mg/kg within the 10-month-old group (*p* = 0·9).

Plaque development was assessed in these mice (Fig. 7A,D,F&I). While the percentage plaque coverage of the hippocampus was not significantly different between the doses (one-way ANOVA, *App*^NL-G-F^: *p* = 0·3; *App*^NL-F^: *p* = 0·6) nor was the density of plaques within the hippocampal region per section (*App*^NL-G-F^: *p* = 0·6; *App*^NL-F^: *p* = 0·5), there was a change in the density of small plaques in *App*^NL-G-F^ mice (Fig. 7D). In *App*^NL-G-F^, there was a significant effect of plaque size (*p* < 0·0001; repeated measures two-way ANOVA), simply reflecting more small than large plaques. While there was no main effect of drug (*p* = 0·1), there was a significant size × drug interaction (p = 0·0040). Dunnett’s comparisons within plaque-size bins showed significant effects of both 300 mg/kg (*p* = 0·0002) and 1200 mg/kg (*p* < 0·0001) PLX5622 for plaques up to 200 μm^2^ but not for larger plaques. In *App*^NL-F^ mice, there was a significant main effect of size (*p* < 0.0001) but no effects of dose (*p* = 0·9) or interaction (*p* = 0·9; Fig. 7I).

### Microglia ablation – synaptic transmission

The effects of PLX5622 on paired-pulse ratios in 3·5-month-old animals (Fig. 7E) were assessed using two-way ANOVAs within inter-stimulus interval. There was a significant main effect of drug (*p* = 0·018) at 25 ms but no effect of genotype (*p* = 0·3) or dose × genotype interaction (*p* = 0·3). Simple Sidak-corrected comparisons of drug dose indicated that 300 mg/kg (*p* = 0·026) but not 1200 mg/kg PLX5622 (*p* > 0·5) was significantly different from control; 300 mg/kg versus 1200 mg/kg PLX5622 reached *p* = 0·075. There were no significant differences at the 50 ms interval.

In 10-month-old mice, the low-dose PLX5622 markedly reduced paired-pulse ratios, preventing paired-pulse facilitation in wild type and exacerbating the reduced ratios in *App*^NL-F^ mice (Fig. 7J). The high-dose PLX5622 had little effect in either genotype. A two-way ANOVA within the 25 ms inter-stimulus interval revealed a significant main effect of dose (*p* < 0.0002) but not genotype (*p* = 0·4) and a significant dose × genotype interaction (*p* = 0·036). Simple Sidak-corrected comparisons within drug indicated that 300 mg/kg (*p* < 0·0001) but not 1200 mg/kg PLX5622 (*p* > 0·2) was significantly different from control; the significance of the difference between 300 mg/kg and 1200 mg/kg PLX5622 reached *p* = 0·02. At the 50 ms inter-stimulus interval, there was a significant main effect of dose (p = 0·024) but no effect of genotype (*p* = 0·2) or dose × genotype interaction (*p* = 0·7). Simple comparisons of PLX5622 dose with Sidak post hoc corrections indicated that the significance of the difference between control and low-dose PLX5622 reached *p* = 0·020. (Neither control versus high-dose, or low-dose versus high-dose were significant, *p* = 0·4.)

To test that changing our standard grain-based mouse diet to the refined diet in which PLX5622 was provided did not unexpectedly change paired-pulse ratios, we compared data presented in Fig. 3 to the control diet. The change of diet had no significant effect on paired-pulse ratios in either genotype (Supplementary Fig. 4). Thus, the lower paired-pulse ratio in 9-month-old *App*^NL-F^ mice was confirmed in an independent 10-month-old cohort fed the refined diet.

Thus, partial removal of microglia, more so than total ablation, renders paired-pulse ratios in older wild types similar to those of *App* knock-ins and, furthermore, exacerbates the *App* knock-in phenotype without altering the plaque load of the mice.

## Discussion

**Fig. 8:**
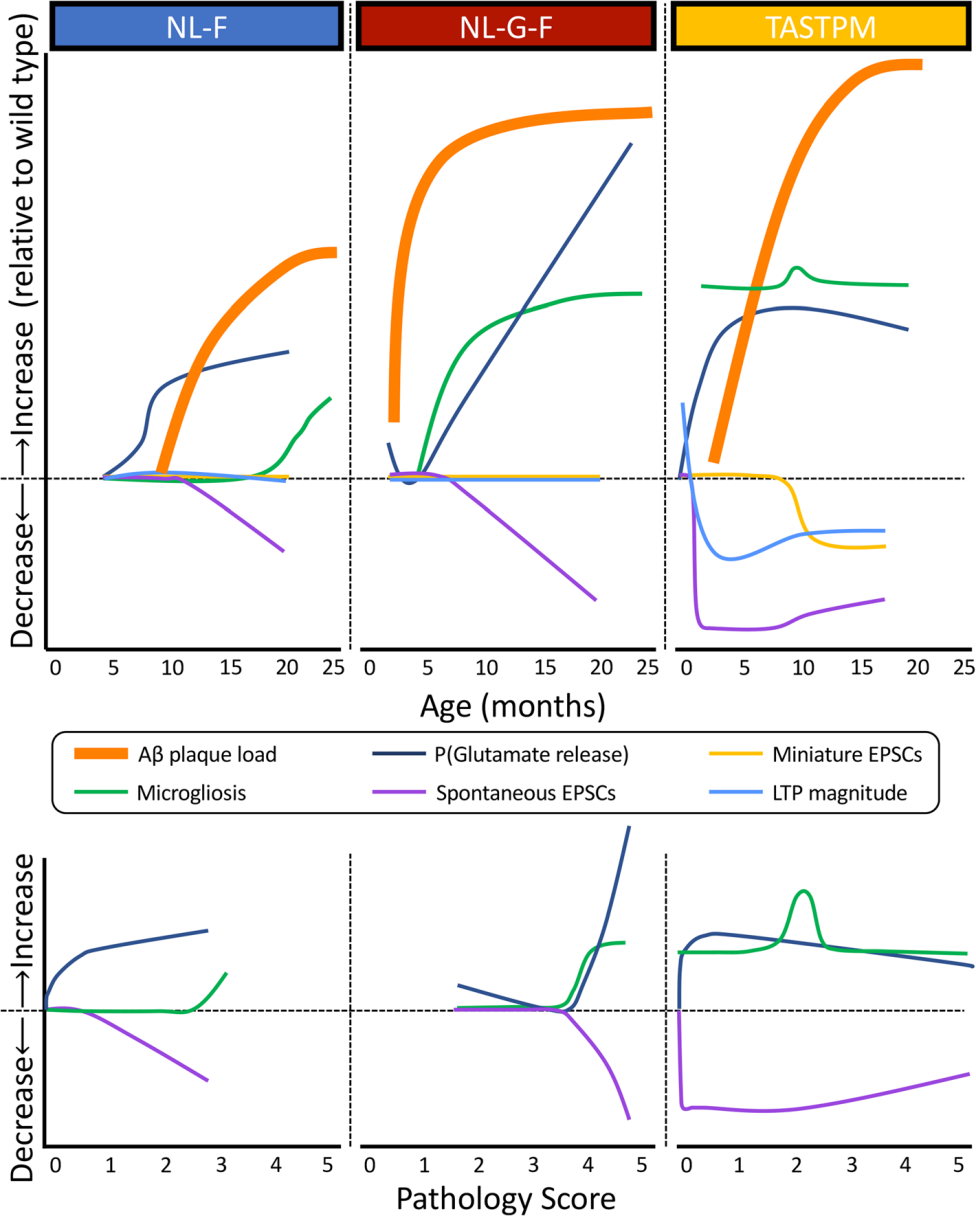
Summary timelines of phenotypes in APP knock-in and transgenic mice. Timelines for indicated phenotypes in *App*^NL-F^ (left), *App*^NL-G-F^ (centre) and TASTPM transgenic mice (right). *App*^NL-F^ and *App*^NL-G-F^ data are all included in the current publication. TASTPM data were published in Matarin et al., 2014; Cummings et al., 2015; and Medawar et al., 2019 (a preliminary comparison was presented in Joel et al., 2018). Note the phenotypic development in relation to plaque deposition within each mouse model and across the three models. Magnitudes of change across different phenotypes are not necessarily proportional; however, within any given phenotype, magnitudes of change across genotypes are proportional. Microgliosis refers to densities of IBA1^+^ microglia. Probability of glutamate release is based on paired-pulse ratio data. Spontaneous and miniature EPSCs reflect changes in frequency

*App* knock-in mice avoid many problems associated with transgenic models, particularly overexpression and inappropriate expression driven by non-endogenous promoters. Importantly, when these confounding factors are removed, the key phenotypes observed in TASTPM (*APP*_Swe_/*PSEN1*_M146V_) transgenic mice from our previous studies are maintained [9, 10]; see also [27]. However, the timing and magnitude of these phenotypes are different, both across transgenic and knock-in models and between the two knock-in models (summarised in Fig. 8). Also of note is that we find little evidence for differential effects between sexes in either microglial proliferation or expression of microglial genes.

### Increased probability of release is consistent across mouse models

The simplest interpretation of the decreased paired-pulse ratio is an increased probability of glutamate release [30]. Indeed, at TASTPM CA3-CA1 synapses, we reported a reduction in failures to release neurotransmitter [9] and here we confirm this in the oldest group of *App* knock-in mice. Modulating probability of release appears to be a physiological role of Aβ [32, 33], which is released in an activity-dependent manner at or near the synapse following endocytosis and processing of APP [34]. In support of the role of synaptic modulation, conditional knockout of *App* indeed reduced probability of glutamate release as mouse CA3-CA1 synapses, likely reflecting the role of Aβ, although other APP metabolites could also mediate the effect [35]. Extensive reviews of the controversial hypotheses surrounding the regulation of neurotransmitter release in Alzheimer’s disease are provided in references [36, 37].

Two mechanisms by which Aβ may increase the probability of glutamate release are pertinent: Increased synapsin 1 phosphorylation following enhanced Ca^++^ influx and calcium/calmodulin kinase IV activity, which results in greater synaptic vesicle availability [38, 39]; and disruption of the interaction of synaptophysin 1 with VAMP2, thus increasing the availability of VAMP2 to participate in the SNARE complex and mediate neurotransmitter release [40]. Interestingly, gene expression of other SNARE proteins are altered in mouse models of Alzheimer’s disease, including in our previous data published on www.mouseac.org. For example, *Snap25* is reduced in TASTPM mice. A halving of SNAP25 using siRNA in immature primary hippocampal neurones, rather than impairing neurotransmitter release, unexpectedly results in a shift from paired-pulse facilitation to paired-pulse depression [41]. Thus, while SNAP25 is critical for neurotransmitter release, partial reduction can result in an increase in probability of release. Other presynaptic markers, including *Syt1* and *Syp* (encoding synaptotagmin-1 and synaptophysin, respectively), show reductions in expression in TASTPM transgenic mice (www.mouseac.org) and also in patients [42, 43]. While a reduction in any one of these proteins may be predicted to impair vesicle release, the complex phenotype associated with variable reductions in these and the SNARE proteins is harder to predict. Indeed, multiple knock-out of similar genes results in an increased release probability [44], similar to that observed here.

Thus, increased release probability is common to transgenic and knock-in models and starts before plaque deposition (Fig. 8), presumably in response to rising soluble Aβ levels. The loss of this effect in *App*^NL-G-F^ mice with a moderate plaque load and the later observation that *App*^NL-F^ and *App*^NL-G-F^ mice show similar magnitude effects despite a heavier plaque load in *App*^NL-G-F^ mice, likely reflect the strong equilibrium shift towards Aβ deposition caused by the Arctic mutation [25]. Indeed *App*^NL-G-F^ mice have lower soluble Aβ levels than *App*^NL-F^ mice, despite heavier plaque loads [21]. As soluble Aβ is the more likely mediator of synaptic modulation, it is less surprising that removing soluble Aβ from the neuropil (caused by the Arctic mutation sequestering it into plaques) results in an alleviation of the phenotype. Presumably, there is always some soluble Aβ in the immediate vicinity of the plaque, even in *App*^NL-G-F^ mice, resulting in a return of the phenotype when the plaque load across the whole neuropil is heavy enough for most synapses to be within this close range.

### Temporal differences in loss of spontaneous action potentials between models

The loss of spontaneous action potentials (i.e. spontaneous EPSCs), identified before plaque deposition in transgenic mice, is much later in *App* knock-in mice. In *App*^NL-G-F^ mice, this only occurs from 9-months-old, when the plaque load is very heavy and coinciding with the re-emergence of altered paired-pulse ratios. In *App*^NL-F^ mice, this loss is even later, at 20-months-old and much later than changes in release probability. While the plaque load is quite heavy at this stage, it is substantially less than in the 9-months-old *App*^NL-G-F^ mice. This is also consistent with an effect of soluble Aβ affecting synapses more distal from plaques in the *App*^NL-F^ than occurs in *App*^NL-G-F^ mice. The loss of spontaneous synaptic activity prior to plaque deposition in transgenic mice likely reflects the much higher levels of soluble Aβ in these APP overexpressing mice. Avoiding overexpression has resulted in a temporal separation of the increased release probability (occurring prior to plaque deposition) and loss of spontaneous action potentials (only occurring once a substantial plaque load is evident) in the knock-in mice, which were concomitant in the transgenic animals and preceded plaque deposition. Thus, it may be that the loss of spontaneous action potentials is a homeostatic response to the increased glutamate release; it certainly is not the reverse (see [45] for review). Importantly, this consistency of phenotypes between models suggests that increased release probability is likely the earliest effect of rising Aβ levels.

We do not see the increase in probability of release, indicated by the decreased paired-pulse ratio and reduced failure rate, reflected as an increase in frequency of miniature (or spontaneous) EPSCs. This may reflect that the increase in probability of release is counteredbalanced by a reduction in the number of synapses, supported by a reduction in synaptophysin and PSD95 immunoreactivity in *App*^NL-F^ mice from 9 months of age [21]. Alternatively, it would be pertinent to investigate differences in vesicle pools that underlie the various forms of synaptic transmission. The immediately releasable pool underlies synchronous release of neurotransmitter, i.e. glutamate release in response to an action potential, and typically consists of a single vesicle at hippocampal synapses and thus results in quantal (i.e. single vesicle) release [46]. In contrast, asynchronous release, i.e. glutamate release underlying the stochastic miniature currents, arises from a pool of vesicles that are very close to but not fully primed for release, referred to as the readily releasable pool [46]. Thus, our data indicate that regulation of the immediately releasable pool may be altered, while the readily releasable pool may be unaffected.

The increase in release probability due to soluble Aβ, even before plaque deposition is consistent with observations in vivo in transgenic mice of increased activity in many neurones in the network (reviewed in [47]). Moreover, this effect is shown to persist in these in vivo studies especially in the vicinity of plaques where soluble Aβ would be expected to be highest. Although we do not see an increase in spontaneous (including miniature) glutamatergic activity in the present study, nor in our previous study in transgenic mice, this is likely a difference between in vivo recordings at physiological temperatures with the full neuronal network intact versus in vitro acute brain slice recordings at room temperature, in which much of the recurrent activity is likely to be truncated. Moreover, the present recordings were in the presence of a GABAergic antagonist, which greatly decreased the effects reported in vivo. It should, however, be noted that we do not see alterations in spontaneous GABAergic IPSCs, either here in *App* knock-in mice, or previously in transgenic mice [10], which is consistent with the observation that palvalbumin-positive interneurones, despite showing some neuritic dystrophy, are otherwise normal in *App*^NL-F^ mice [48]. Thus, we would not suggest that we could assess the overall physiological network effects of Aβ in the present study but can be confident about the changes seen in individual synapses. It would be interesting to undertake equivalent in vivo studies in the knock-in mice to address the question of whether these network changes are dependent on overexpression or other artefacts of transgenic technology.

### Synaptic plasticity is intact

Data from transgenic mice suggest that Aβ has a role in the induction and consolidation of LTP, with several labs reporting either its absence or even induction of long-term depression rather than LTP (reviewed in [37]). We have reported a biphasic effect on magnitude of LTP in transgenic mice [10], with an increase in LTP when Aβ levels initially increase but a subsequent reduction in LTP following plaque deposition. Recently it was reported that both early (1–2 h) and late (4 h) LTP is impaired in *App*^NL-G-F^ mice at 6–8 months [49]. However, we do not replicate this phenotype in the knock-in models, even at advanced ages. This discrepancy could be an effect of employing different LTP induction paradigms or Latif-Hernandez and colleagues’ choice of the *App*^*NL*^ knock-in mouse as control, which presents with raised soluble and insoluble Aβ above wild type levels [21] and > 10-fold higher soluble Aβ40 than *App*^NL-G-F^ [49]. Given soluble Aβ40 monomer and dimers increase release probability [33], this complicates the interpretation. While we find no deficits in LTP induction compared to wild type mice, a more complex phenotype is observed, whereby there is a loss of postsynaptic mechanisms of expression counterbalanced by enhanced presynaptic mechanisms as *App*^NL-F^ and *App*^NL-G-F^ mice age. As wild type mice age, a similar but non-significant trend is seen, suggesting the increase in plaque load exacerbates the effect of normal ageing. Notably, all the functional synaptic changes seen in *App* knock-in mice are presynaptic, suggesting that much of the postsynaptic change reported in transgenic mice may be artefacts of transgenic mice.

### Microglia and plaques

Microglial phenotypes have been long-established as part of Alzheimer’s disease pathology. Indeed, Alois Alzheimer noted a glial phenotype alongside plaques and neurofibrillary tangles [50]. More recently, genome-wide association studies highlight that microglial genes are the main groups of risk-genes for Alzheimer’s disease [for example see [51].

In the Alzheimer’s diseased brain, microglia cluster around plaques, which has led to the notion that microglia clear plaques. However, it appears that they are largely ineffective at directly phagocytosing Αβ, both in vitro or in vivo [for example, see [52]. Here we confirm previous reports suggesting that removal of microglia does not increase plaque load [53, 54]; but see the recent article [55]. Indeed, if anything, in our hands there are fewer of the smallest, presumably most recently deposited plaques, when the microglia are removed with a CSF1R inhibitor. This is consistent with microglia moderating the initial development of plaques [28] but, as we report the same density and coverage of plaques at early stages, microglia probably do not mediate seeding of plaques. We recently proposed that it is more likely that synaptic toxicity imparted by Aβ induces microglia to phagocytose damaged synapses rather than Aβ, limiting the spread of damage [18]. Supporting the suggestion that microglia phagocytose synapses, a recent prepublication shows adult human microglia containing synaptic proteins and that synapses derived from Alzheimer’s disease brain are phagocytosed more readily [56]. Alternative roles for microglia in Aβ handling have been proposed, including compacting Aβ via TREM2-dependent mechanisms [57, 58] or clearing Αβ via non-phagocytic mechanisms [summarised in 52].

Interestingly, when microglial numbers were depleted, irrespective of the presence of plaques, the remaining microglia were predominantly CD68^+^. It is not clear whether CD68^+^ microglia are more resistant to CSF1R inhibitors or that the remaining microglia shift towards a phagocytic phenotype. Certainly, in the *App*^NL-G-F^ mice, the density of CD68^+^ microglia was unchanged by the low-dose of PLX5622 compared to untreated mice, despite a halving of the total microglia.

Ablation of microglia exacerbated the decrease in paired-pulse ratio in *App* knock-in mice. This surprising phenotype was more pronounced in response to partial removal of microglia than near-complete ablation, suggesting that the remaining CSF1R-inhibited and mostly CD68^+^ microglia have an active effect, rather than the loss of microglia being the cause of the change. Given that removal of microglia exacerbated synaptic phenotypes in both lines of *App* knock-in mice, microglia are likely protective in mouse models and human disease. It should be noted that 10-month-old wild type mice also responded with a reduced paired-pulse ratio. So, while loss of microglial function may be damaging in Alzheimer’s disease, this is not a disease-specific phenomenon but a change in the same direction, exacerbated in disease. Intriguingly, *Trem2* haploinsufficient mice have a greater microglial response to neuronal injury than wild type mice or those with complete knock-out of the gene. Moreover, *Trem2* haploinsufficiency increases tau pathology, proinflammatory markers and atrophy in transgenic mutant human tau mice. Conversely, complete *Trem2* deficiency is protective in the tau mice [59].

If reactive microglia do indeed mediate some of the phenotypes mediated here, there are several routes by which they may exert effects. One is a direct interaction of microglia and neurones, which the shift towards a CD68^+^ profile suggests as being increased phagocytosis. As already discussed, we suggest that this is phagocytosis of damaged synapses surrounding plaques, rather than of plaques per se [18] and that this limits the spread of damage but may later become detrimental as the number of synapses (or, indeed, neurites) excised damage overall network activity. In parallel to this, it has been proposed that the profiles of microglia change during disease progress, in particular there is shift in the gene profiles towards more proinflammatory markers [60]. This shift is reflected in the *App* knock-in mice [61] (which is also true for transgenic mice, e.g. [13, 15, 62]). In addition, the neuronally-released cytokine IL33, has been shown to regulate microglia such that they promote structural synaptic plasticity by remodelling the extracellular matrix and promotes fear conditioned learning [63]. Extending that role into a transgenic mouse model of Alzheimer’s disease, IL33 shifted the activation state of microglia from a proinflammatory profile towards a profile expressing anti-inflammatory genes with improved phagocytosis and again improved fear-conditioned learning [64]. Thus, it appears that microglia can directly modulate synaptic structure, function and, ultimately, behaviour of the animal, particularly during disease states.

### Contrasting phenotypes in the *App* knock-in mice is likely due to the Arctic mutation

The lack of electrophysiological and microglial phenotypes in the *App*^NL-G-F^ mice when plaques first appear is in contrast to the clear effects in both transgenic mice [9, 10] and *App*^NL-F^ mice at an equivalent stage of Aβ pathology. This cannot be an effect specific to 4-month-old animals because the transgenic mice maintain microgliosis and altered release probability at this age (Fig. 8). Again, lower soluble Aβ levels due to the higher propensity for Aβ fibrillation imparted by the Arctic mutation may be the cause. So, while the *App*^NL-G-F^ model is attractive as it presents with rapid plaque formation, its usefulness as a model for Alzheimer’s disease is in understanding how folding of Aβ and proximity to plaques affect phenotypes. In contrast, the mutations in *App*^NL-F^ mice are restricted to the β- and γ-secretase cleavage sites flanking the Aβ sequence and are thus used as a tool to trigger the raise in Aβ levels observed in sporadic Alzheimer’s disease. Importantly, despite the more rapid plaque deposition in *App*^NL-G-F^, phenotypes are more pronounced and at earlier Aβ pathology stages in the *App*^NL-F^ mice.

Another consideration in comparing models is the choice of control. Until now the wild type mouse has been the best available control but this has its limitations. The difference in sequence between human and mouse APP alters the affinity for β-secretase. Humanising *App* increases levels of Aβ compared to wild type mice [65], suggesting that even with knock-in mice, some of the changes may be exaggerated when compared to wild types. The humanised *App* mouse will be an improved control in future studies.

Given that Aβ is a physiological protein, its basal effects are likely beneficial. Indeed, the proposed overall physiological function of Aβ is to improve memory [66]. We propose that increased neurotransmitter release mediated by Aβ is a protective response to events such as head trauma, ischaemia or other synaptic insults. If these insults are short-lived, Aβ levels return to normal, restoring neuronal activity. However, in individuals with either impaired Aβ clearance, deficient immune function or in which the cause of the Aβ elevation persists, for example in chronic conditions such as high-blood pressure and type II diabetes (both risk factors for Alzheimer’s disease), Aβ levels will remain raised. Extended periods of high Aβ levels will maintain altered synaptic function and potential to homeostatic responses and lead to plaque deposition, tau hyperphosphorylation and dystrophic neurites. In mice, this is the extent of pathology; however, humans progress to neurofibrillary tangles and neurodegeneration. The *App*^NL-F^ mice provide a background of rising Aβ which likely mimics early stages of disease without the artefacts of transgenic mice and provides a good base model for the addition of further risk-factors that may lead to improvements in the available models and understanding of the disease.

## Conclusions

Increased probability of glutamate release precedes plaque deposition, is consistent across transgenic and knock-in mouse models of Alzheimer’s disease and likely reflects the acute physiological effect of Aβ. Inappropriately prolonged increases in glutamate release maintains aberrant synaptic transmission and thus may result in homeostatic changes, such as the decrease in action potential firing observed here. Given the positive regulation of glutamate release by activity-dependent release of Aβ [32], a positive feed-back may be triggered by dysregulated Aβ release or clearance. Ever increasing levels of amyloid beta would lead to deposition and subsequent damage around plaques [67]. However, the effects of plaques may well depend on their structure, as indicated by the differential time course of *App*^NL-G-F^ compared to *App*^NL-F^ at similar plaque loads, which likely reflects, in this instance, the Acrtic mutation pushing the Aβ equilibrium from soluble towards insoluble forms, creating more dense plaques. However, differences in structure may also exist within sporadic forms of the disease and thus variations in the halo of oligomeric forms of Aβ surrounding plaques [68] may influence their effects on synaptic function or toxicity.

Microglia also respond to increased Aβ levels by proliferating and upregulating *CD68* and *Trem2*. Partial depletion of microglia indicated that the surviving phagocytic microglia, rather than their absence, likely drive the age-dependent effect on glutamate release that becomes exacerbated in Alzheimer’s disease.

We also shed light on which of the previous reports from transgenic mice can be validated in knock-in mice. This is the first detailed characterisation and comparison of the relationship between plaques, synaptic transmission and microglial activity throughout life in the two *App* knock-in mouse lines *App*^NL-F^ and *App*^NL-G-F^, in which overexpression and other artefacts associated with transgenic technology are avoided.

## Supplementary Information

**Additional file 1.** Expanded Methods.

**Additional file 2.Supplementary figure 1.** Plaque visual grading reference images.

**Additional file 3.Supplementary figure 2.** Plaque development increases with age in *App*^NL-F^ and *App*^NL-G-F^ mice (300 μm sections).

**Additional file 4.Supplementary figure 3.** Spontaneous inhibitory postsynaptic currents are unaltered.

**Additional file 5.Supplementary figure 4.** Field EPSP input-output relationships are normal.

**Additional file 6.Supplementary figure 5.** Changing diet from grain-based to refined formulations has no effect on paired-pulse ratios.

## Data Availability

The datasets used and/or analysed during the current study are available from the corresponding author on reasonable request.

## Abbreviations

Aβ: amyloid beta
aCSF: artificial cerebrospinal fluid
EPSC: excitatory postsynaptic current
fEPSP: field excitatory postsynaptic potential
GD: grain-based diet
GLMM: generalised linear mixed model
LCOs: luminescent conjugated oligothiophenes
NS: not significant
LTP: long-term potentiation
RD: refined diet
RT-qPCR: real time quantitative PCR

## References

[1] Gotz J, Bodea LG, Goedert M (2018). Rodent models for Alzheimer disease. Nat Rev Neurosci.

[2] Sasaguri H, Nilsson P, Hashimoto S, Nagata K, Saito T, De Strooper B, Hardy J, Vassar R, Winblad B, Saido TC (2017). APP mouse models for Alzheimer's disease preclinical studies. EMBO J.

[3] Andrew RJ, Kellett KA, Thinakaran G, Hooper NM (2016). A Greek tragedy: the growing complexity of Alzheimer amyloid precursor protein proteolysis. J Biol Chem.

[4] Rice HC, de Malmazet D, Schreurs A, Frere S, Van Molle I, Volkov AN, et al. Secreted amyloid-beta precursor protein functions as a GABABR1a ligand to modulate synaptic transmission. Science. 2019;363:eaao4827. 10.1126/science.aao4827. 10.1126/science.aao4827 PMC6366617 30630900

[5] Garcia-Gonzalez L, Pilat D, Baranger K, Rivera S (2019). Emerging alternative proteinases in APP metabolism and Alzheimer's disease pathogenesis: a focus on MT1-MMP and MT5-MMP. Front Aging Neurosci.

[6] Tozzi A, Sclip A, Tantucci M, de Iure A, Ghiglieri V, Costa C, Di Filippo M, Borsello T, Calabresi P (2015). Region- and age-dependent reductions of hippocampal long-term potentiation and NMDA to AMPA ratio in a genetic model of Alzheimer's disease. Neurobiol Aging.

[7] Jacobsen JS, Wu CC, Redwine JM, Comery TA, Arias R, Bowlby M, Martone R, Morrison JH, Pangalos MN, Reinhart PH, Bloom FE (2006). Early-onset behavioral and synaptic deficits in a mouse model of Alzheimer's disease. Proc Natl Acad Sci U S A.

[8] D'Amelio M, Cavallucci V, Middei S, Marchetti C, Pacioni S, Ferri A, Diamantini A, De Zio D, Carrara P, Battistini L (2011). Caspase-3 triggers early synaptic dysfunction in a mouse model of Alzheimer's disease. Nat Neurosci.

[9] Cummings DM, Liu W, Portelius E, Bayram S, Yasvoina M, Ho SH, Smits H, Ali SS, Steinberg R, Pegasiou CM, James OT, Matarin M, Richardson JC, Zetterberg H, Blennow K, Hardy JA, Salih DA, Edwards FA (2015). First effects of rising amyloid-beta in transgenic mouse brain: synaptic transmission and gene expression. Brain.

[10] Medawar E, Benway TA, Liu W, Hanan TA, Haslehurst P, James OT, Yap K, Muessig L, Moroni F, Nahaboo Solim MA, Baidildinova G, Wang R, Richardson JC, Cacucci F, Salih DA, Cummings DM, Edwards FA (2019). Effects of rising amyloidbeta levels on hippocampal synaptic transmission, microglial response and cognition in APPSwe/PSEN1M146V transgenic mice. EBioMedicine.

[11] Condello C, Yuan P, Schain A, Grutzendler J (2015). Microglia constitute a barrier that prevents neurotoxic protofibrillar Abeta42 hotspots around plaques. Nat Commun.

[12] Hong S, Beja-Glasser VF, Nfonoyim BM, Frouin A, Li S, Ramakrishnan S, Merry KM, Shi Q, Rosenthal A, Barres BA, Lemere CA, Selkoe DJ, Stevens B (2016). Complement and microglia mediate early synapse loss in Alzheimer mouse models. Science.

[13] Sala Frigerio C, Wolfs L, Fattorelli N, Thrupp N, Voytyuk I, Schmidt I, Mancuso R, Chen WT, Woodbury ME, Srivastava G, Möller T, Hudry E, Das S, Saido T, Karran E, Hyman B, Perry VH, Fiers M, de Strooper B (2019). The Major risk factors for Alzheimer's disease: age, sex, and genes modulate the microglia response to Abeta plaques. Cell Rep.

[14] Kunkle BW, Grenier-Boley B, Sims R, Bis JC, Damotte V, Naj AC, Boland A, Vronskaya M, van der Lee SJ, Amlie-Wolf A (2019). Genetic meta-analysis of diagnosed Alzheimer's disease identifies new risk loci and implicates Abeta, tau, immunity and lipid processing. Nat Genet.

[15] Matarin M, Salih DA, Yasvoina M, Cummings DM, Guelfi S, Liu W, Nahaboo Solim MA, Moens TG, Paublete RM, Ali SS, Perona M, Desai R, Smith KJ, Latcham J, Fulleylove M, Richardson JC, Hardy J, Edwards FA (2015). A genome-wide gene-expression analysis and database in transgenic mice during development of amyloid or tau pathology. Cell Rep.

[16] Salih DA, Bayram S, Guelfi S, Reynolds RH, Shoai M, Ryten M, et al. Genetic variability in response to amyloid beta deposition influences Alzheimer's disease risk. Brain Commun. 2019;1:fcz022. 10.1093/braincomms/fcz022. 10.1093/braincomms/fcz022 PMC7145452 32274467

[17] Sierksma A, Lu A, Mancuso R, Fattorelli N, Thrupp N, Salta E, et al. Novel Alzheimer risk genes determine the microglia response to amyloid-beta but not to TAU pathology. EMBO Mol Med. 2020;12:e10606. 10.15252/emmm.201910606. 10.15252/emmm.201910606 PMC7059012 31951107

[18] Edwards FA (2019). A unifying hypothesis for Alzheimer's disease: from plaques to Neurodegeneration. Trends Neurosci.

[19] Hong S, Dissing-Olesen L, Stevens B (2016). New insights on the role of microglia in synaptic pruning in health and disease. Curr Opin Neurobiol.

[20] Spangenberg EE, Green KN (2017). Inflammation in Alzheimer's disease: lessons learned from microglia-depletion models. Brain Behav Immun.

[21] Saito T, Matsuba Y, Mihira N, Takano J, Nilsson P, Itohara S, Iwata N, Saido TC (2014). Single app knock-in mouse models of Alzheimer's disease. Nat Neurosci.

[22] Forman MS, Cook DG, Leight S, Doms RW, Lee VM (1997). Differential effects of the swedish mutant amyloid precursor protein on beta-amyloid accumulation and secretion in neurons and nonneuronal cells. J Biol Chem.

[23] Guardia-Laguarta C, Pera M, Clarimon J, Molinuevo JL, Sanchez-Valle R, Llado A, Coma M, Gomez-Isla T, Blesa R, Ferrer I, Lleo A (2010). Clinical, neuropathologic, and biochemical profile of the amyloid precursor protein I716F mutation. J Neuropathol Exp Neurol.

[24] Karran E, Mercken M, De Strooper B (2011). The amyloid cascade hypothesis for Alzheimer's disease: an appraisal for the development of therapeutics. Nat Rev Drug Discov.

[25] Nilsberth C, Westlind-Danielsson A, Eckman CB, Condron MM, Axelman K, Forsell C, Stenh C, Luthman J, Teplow DB, Younkin SG, Näslund J, Lannfelt L (2001). The 'Arctic' APP mutation (E693G) causes Alzheimer's disease by enhanced Abeta protofibril formation. Nat Neurosci.

[26] Saito T, Mihira N, Matsuba Y, Sasaguri H, Hashimoto S, Narasimhan S, Zhang B, Murayama S, Higuchi M, Lee VMY, Trojanowski JQ, Saido TC (2019). Humanization of the entire murine Mapt gene provides a murine model of pathological human tau propagation. J Biol Chem.

[27] Joel Z, Izquierdo P, Salih DA, Richardson JC, Cummings DM, Edwards FA (2018). Improving Mouse Models for Dementia. Are all the effects in tau mouse models due to overexpression?. Cold Spring Harb Symp Quant Biol.

[28] Spangenberg E, Severson PL, Hohsfield LA, Crapser J, Zhang J, Burton EA, Zhang Y, Spevak W, Lin J, Phan NY, Habets G, Rymar A, Tsang G, Walters J, Nespi M, Singh P, Broome S, Ibrahim P, Zhang C, Bollag G, West BL, Green KN (2019). Sustained microglial depletion with CSF1R inhibitor impairs parenchymal plaque development in an Alzheimer's disease model. Nat Commun.

[29] Nystrom S, Back M, Nilsson KPR, Hammarstrom P. Imaging amyloid tissues stained with luminescent conjugated Oligothiophenes by Hyperspectral confocal microscopy and fluorescence lifetime imaging. J Vis Exp. 2017;(128). 10.3791/56279. 10.3791/56279 PMC5755170 29155738

[30] Zucker RS, Regehr WG (2002). Short-term synaptic plasticity. Annu Rev Physiol.

[31] Bliss TV, Collingridge GL (2013). Expression of NMDA receptor-dependent LTP in the hippocampus: bridging the divide. Molecular brain.

[32] Abramov E, Dolev I, Fogel H, Ciccotosto GD, Ruff E, Slutsky I (2009). Amyloid-beta as a positive endogenous regulator of release probability at hippocampal synapses. Nat Neurosci.

[33] Fogel H, Frere S, Segev O, Bharill S, Shapira I, Gazit N, O'Malley T, Slomowitz E, Berdichevsky Y, Walsh DM (2014). APP homodimers transduce an amyloid-beta-mediated increase in release probability at excitatory synapses. Cell Rep.

[34] Cirrito JR, Kang JE, Lee J, Stewart FR, Verges DK, Silverio LM, Bu G, Mennerick S, Holtzman DM (2008). Endocytosis is required for synaptic activity-dependent release of amyloid-beta in vivo. Neuron.

[35] Lee SH, Kang J, Ho A, Watanabe H, Bolshakov VY, Shen J (2020). APP family regulates neuronal excitability and synaptic plasticity but not neuronal survival. Neuron.

[36] Marsh J, Alifragis P (2018). Synaptic dysfunction in Alzheimer's disease: the effects of amyloid beta on synaptic vesicle dynamics as a novel target for therapeutic intervention. Neural Regen Res.

[37] Ricciarelli R, Fedele E (2018). cAMP, cGMP and amyloid beta: three ideal Partners for Memory Formation. Trends Neurosci.

[38] Marsh J, Bagol SH, Williams RSB, Dickson G, Alifragis P. Synapsin I phosphorylation is dysregulated by beta-amyloid oligomers and restored by valproic acid. Neurobiol Dis. 2017;106:63–75. 10.1016/j.nbd.2017.06.011. 10.1016/j.nbd.2017.06.011 28647556

[39] Park D, Na M, Kim JA, Lee U, Cho E, Jang M, Chang S: Activation of CaMKIV by soluble amyloid-beta1-42 impedes trafficking of axonal vesicles and impairs activity-dependent synaptogenesis. Sci Signal. 2017;10:eaam8661. 10.1126/scisignal.aam8661. 10.1126/scisignal.aam8661 28698220

[40] Russell CL, Semerdjieva S, Empson RM, Austen BM, Beesley PW, Alifragis P (2012). Amyloid-beta acts as a regulator of neurotransmitter release disrupting the interaction between synaptophysin and VAMP2. PLoS One.

[41] Antonucci F, Corradini I, Morini R, Fossati G, Menna E, Pozzi D, Pacioni S, Verderio C, Bacci A, Matteoli M (2013). Reduced SNAP-25 alters short-term plasticity at developing glutamatergic synapses. EMBO Rep.

[42] Masliah E, Mallory M, Alford M, DeTeresa R, Hansen LA, McKeel DW, Morris JC (2001). Altered expression of synaptic proteins occurs early during progression of Alzheimer's disease. Neurology.

[43] Yao PJ, Zhu M, Pyun EI, Brooks AI, Therianos S, Meyers VE, Coleman PD (2003). Defects in expression of genes related to synaptic vesicle trafficking in frontal cortex of Alzheimer's disease. Neurobiol Dis.

[44] Raja MK, Preobraschenski J, Del Olmo-Cabrera S, Martinez-Turrillas R, Jahn R, Perez-Otano I, et al. Elevated synaptic vesicle release probability in synaptophysin/gyrin family quadruple knockouts. Elife. 2019;8. 10.7554/eLife.40744. 10.7554/eLife.40744 PMC6519982 31090538

[45] Styr B, Slutsky I (2018). Imbalance between firing homeostasis and synaptic plasticity drives early-phase Alzheimer's disease. Nat Neurosci.

[46] Hagler DJ, Goda Y (2001). Properties of synchronous and asynchronous release during pulse train depression in cultured hippocampal neurons. J Neurophysiol.

[47] Busche MA, Konnerth A (2015). Neuronal hyperactivity--a key defect in Alzheimer's disease?. Bioessays.

[48] Sos KE, Mayer MI, Takacs VT, Major A, Bardoczi Z, Beres BM, Szeles T, Saito T, Saido TC, Mody I (2020). Amyloid beta induces interneuron-specific changes in the hippocampus of APPNL-F mice. PLoS One.

[49] Latif-Hernandez A, Sabanov V, Ahmed T, Craessaerts K, Saito T, Saido T, Balschun D (2020). The two faces of synaptic failure in app (NL-G-F) knock-in mice. Alzheimers Res Ther.

[50] Strassnig M, Ganguli M (2005). About a peculiar disease of the cerebral cortex: Alzheimer's original case revisited. Psychiatry (Edgmont).

[51] Neuner SM, Tcw J, Goate AM (2020). Genetic architecture of Alzheimer's disease. Neurobiol Dis.

[52] Fu H, Liu B, Li L, Lemere CA (2020). Microglia do not take up soluble amyloid-beta peptides, but partially degrade them by secreting insulin-degrading enzyme. Neuroscience.

[53] Spangenberg EE, Lee RJ, Najafi AR, Rice RA, Elmore MR, Blurton-Jones M, West BL, Green KN (2016). Eliminating microglia in Alzheimer's mice prevents neuronal loss without modulating amyloid-beta pathology. Brain.

[54] Olmos-Alonso A, Schetters ST, Sri S, Askew K, Mancuso R, Vargas-Caballero M, Holscher C, Perry VH, Gomez-Nicola D (2016). Pharmacological targeting of CSF1R inhibits microglial proliferation and prevents the progression of Alzheimer's-like pathology. Brain.

[55] Clayton K, Delpech JC, Herron S, Iwahara N, Ericsson M, Saito T, Saido TC, Ikezu S, Ikezu T: Plaque associated microglia hyper-secrete extracellular vesicles and accelerate tau propagation in a humanized APP mouse model. Mol Neurodegener. 2021;16:18. 10.1186/s13024-021-00440-9. 10.1186/s13024-021-00440-9 PMC7986521 33752701

[56] Tzioras M, Daniels MJD, King D, Popovic K, Holloway RK, Stevenson AJ, et al. Altered synaptic ingestion by human microglia in Alzheimer’s disease. bioRxiv. 2019;ID795930:1–25. 10.1101/795930.

[57] Yuan P, Condello C, Keene CD, Wang Y, Bird TD, Paul SM, Luo W, Colonna M, Baddeley D, Grutzendler J (2016). TREM2 Haplodeficiency in mice and humans impairs the microglia barrier function leading to decreased amyloid compaction and severe axonal dystrophy. Neuron.

[58] Wang Y, Ulland TK, Ulrich JD, Song W, Tzaferis JA, Hole JT, Yuan P, Mahan TE, Shi Y, Gilfillan S, Cella M, Grutzendler J, DeMattos RB, Cirrito JR, Holtzman DM, Colonna M (2016). TREM2-mediated early microglial response limits diffusion and toxicity of amyloid plaques. J Exp Med.

[59] Sayed FA, Telpoukhovskaia M, Kodama L, Li Y, Zhou Y, Le D, Hauduc A, Ludwig C, Gao F, Clelland C (2018). Differential effects of partial and complete loss of TREM2 on microglial injury response and tauopathy. Proc Natl Acad Sci U S A.

[60] Leng F, Edison P. Neuroinflammation and microglial activation in Alzheimer disease: where do we go from here? Nat Rev Neurol. 2020;17:157–72. 10.1038/s41582-020-00435-y. 10.1038/s41582-020-00435-y 33318676

[61] Uruno A, Matsumaru D, Ryoke R, Saito R, Kadoguchi S, Saigusa D, et al. Nrf2 suppresses oxidative stress and inflammation in app Knock-in Alzheimer's disease model mice. Mol Cell Biol. 2020;40(6):e00467–19. 10.1128/MCB.00467-19. 10.1128/MCB.00467-19 PMC7048263 31932477

[62] Keren-Shaul H, Spinrad A, Weiner A, Matcovitch-Natan O, Dvir-Szternfeld R, Ulland TK, David E, Baruch K, Lara-Astaiso D, Toth B, Itzkovitz S, Colonna M, Schwartz M, Amit I (2017). A unique microglia type associated with restricting development of Alzheimer's disease. Cell.

[63] Nguyen PT, Dorman LC, Pan S, Vainchtein ID, Han RT, Nakao-Inoue H, Taloma SE, Barron JJ, Molofsky AB, Kheirbek MA, Molofsky AV (2020). Microglial remodeling of the extracellular matrix promotes synapse plasticity. Cell.

[64] Fu AK, Hung KW, Yuen MY, Zhou X, Mak DS, Chan IC, Cheung TH, Zhang B, Fu WY, Liew FY, Ip NY (2016). IL-33 ameliorates Alzheimer's disease-like pathology and cognitive decline. Proc Natl Acad Sci U S A.

[65] Serneels L, T'Syen D, Perez-Benito L, Theys T, Holt MG, De Strooper B (2020). Modeling the beta-secretase cleavage site and humanizing amyloid-beta precursor protein in rat and mouse to study Alzheimer's disease. Mol Neurodegener.

[66] Morley JE, Farr SA, Nguyen AD, Xu F (2019). Editorial: what is the physiological function of amyloid-Beta protein?. J Nutr Health Aging.

[67] Wu HY, Hudry E, Hashimoto T, Kuchibhotla K, Rozkalne A, Fan Z, Spires-Jones T, Xie H, Arbel-Ornath M, Grosskreutz CL, Bacskai BJ, Hyman BT (2010). Amyloid beta induces the morphological neurodegenerative triad of spine loss, dendritic simplification, and neuritic dystrophies through calcineurin activation. J Neurosci.

[68] Querol-Vilaseca M, Colom-Cadena M, Pegueroles J, Nunez-Llaves R, Luque-Cabecerans J, Munoz-Llahuna L, Andilla J, Belbin O, Spires-Jones TL, Gelpi E (2019). Nanoscale structure of amyloid-beta plaques in Alzheimer's disease. Sci Rep.

