# Supplementary material for: Knock-in models related to Alzheimer’s disease: synaptic transmission, plaques and the role of microglia
Source: Mol Neurodegener. 2021 Jul 15;16:47. doi: 10.1186/s13024-021-00457-0 (PMC8281661; doi:10.1186/s13024-021-00457-0)
Supplement: Supplementary file 1 — Additional file 1. Expanded Methods. [file 13024_2021_457_MOESM1_ESM.docx]

**Expanded Methods**

*Animals*

All experiments were performed in accordance with the UK Animal (Scientific Procedures) Act 1986 and following local ethical review. Homozygous *App* knock in mice were bred in the UCL Biological Services Unit as either heterozygous × heterozygous or homozygous × homozygous colonies. Thus, some wild type counterparts were littermates, while others were provided by a colony of the C57BL/6J background strain housed under the same conditions and bred and maintained at UCL, periodically replenished with new breeding pairs from Charles River Laboratories, UK.

Mice were housed on a 12 h light/12 h dark cycle in individually ventilated cages with *ad libitum* supply of water and a grain-based rodent diet (Envigo 2018 Teklad global 18% protein rodent diet). Mice were housed in same-sex groups of 3-5 mice at weaning and provided with a cardboard shelter and tube, nesting material and wooden chew sticks. Where adult males would otherwise be single-housed due to attrition, a >12-month-old female (i.e. past breeding age) was introduced as a cage mate.

Animals were killed by decapitation and the brain rapidly removed. The brain was bisected and one hemisphere drop-fixed into 10% formalin solution overnight at 4ºC, then subsequently transferred into 30% sucrose, 0·03% sodium azide in 0·01 M phosphate buffered saline (PBS) for storage at 4ºC. The second hemisphere was processed for either electrophysiology or RNA extraction.

*Genotyping*

Tail or ear biopsies were process by either Transnetyx Inc (TN, USA) or an in-house genotyping service at UCL. Processing at UCL is briefly outlined here. Genomic DNA was extracted using the ‘HotSHOT’ lysis method. Alkaline lysis reagent (25 mM NaOH, 0·2 mM EDTA, pH12) was added to tissue samples prior to heating to 95°C for 30 minutes. The sample was then cooled to 4°C before the addition of neutralisation buffer (40 mM Tris-HCl, pH 5). The PCR reaction was performed through addition of MyTaq DNA Polymerase (Bioline) reaction buffer and primer pairs:

5’-ATCTCGGAAGTGAAGATG-3’,

5’-ATCTCGGAAGTGAATCTA-3’,

5’-TGTAGATGAGAACTTAAC-3’ and

5’-CGTATAATGTATGCTATACGAAG-3’

using the cycling parameters: 94°C (30 s), 58°C (30 s), 72°C (30 s), for 30 cycles and a final extension at 72°C for 4 min.

*Plaque detection - luminescent conjugated oligothiophenes*

The drop-fixed hemisphere of each mouse brain was sectioned transverse to the long axis of the hippocampus at either 300 μm using a vibrating microtome (Campden) or 30 μm using a frozen sledge microtome (Leica). Serial sections were stored free-floating in PBS with 0·03% sodium azide at 4°C. Plaques were labelled with luminescent conjugated oligothiophenes (LCOs), a unique class of fluorescent amyloid dyes [1, 2]. Sections were washed three times with PBS followed by labelling with two LCO fluorophores, 2·4 μM q-FTAA and 0·77 μM h-FTAA or the commercially available equivalent Amytracker 520 (1:1000; EBBA Biotech AB, Sweden), for 30 min, in the dark. Sections were then rinsed once with PBS for 10 min, counterstained for nuclei with 4’,6-diamidino-2-phenylindole (DAPI; 1:10,000 in PBS) for 5 min, followed by a final wash in PBS for 10 min. Sections were mounted onto Superfrost Plus glass slides (Fisher) using Fluoromount-G medium (SouthernBiotech), cover-slipped and left to dry before imaging. Photomicrographs of whole hippocampal regions within each section were obtained by area-defined serial scanning using constant light, gain and exposure settings using an epifluorescent EVOS FL Auto Cell Imaging System microscope (Life Technologies) under a 20× objective. LCOs were detected at a wavelength of 488 nm and DAPI at a wavelength of 356 nm, using constant light intensity, gain and exposure settings.

Visual grading was used to score the Aβ pathology for each section. Initially a series of images were used to establish grades 0–5 (Supplementary Fig. 1), representing no plaques through to the heaviest plaque load observed. Each image was then graded independently by two experimenters blinded to genotype (wild type, *App*^NL-F^ or *App*^NL-G-F^), age and sex. Three sections per animal were scored and the average from both experimenters then taken as an animal mean, thus giving rise to scores that fall between the integers 0 – 5.

To determine plaque sizes, a threshold for colour intensity (RGB 0 – 255) in the green channel was determined objectively for each section to account for variable background and staining conditions using ImagePro (Media Cybernetics Inc., version 7.0). A series of plaque size measurements were made across a range of colour intensities that encompassed thresholds that blatantly underestimated and overestimated plaque size. An exponential curve was back-extrapolated from the intensities that over-estimated plaque size and the point where observed data and the exponential deviated was chosen as threshold for measurements. Thus, user-subjective choice of threshold was avoided while permitting variable thresholds across batches of sections and provides a reliable method by which choose thresholds that are consistent across different experimenters. The experimenter was blinded to genotype and drug condition during imaging and thresholding.

*Histology*

Immunohistochemical experiments were performed on tissue drop-fixed in 10% formalin solution. Standard immunohistochemical techniques were employed. A frozen sledge microtome (Leica) was used to make 30 μm sections. Sections were washed in PBS, followed by 0·3% Triton X-100 in phosphate buffer saline and subsequent blocking in 3% goat serum/Triton/PBS for 1 hour. Incubation with primary antibody (IBA1, 1:500, Wako Catalogue number 019-19741; 1:500 rat anti-CD68 Bio-Rad) in blocking solution was performed overnight at 4°C. Alexa-conjugated secondary antibody (1:1000; Invitrogen) was added to the blocking solution for a 2-hour incubation at room temperature in the dark. DAPI (1:10,000) was applied to all sections for 5 minutes prior to mounting. Between all steps, sections were washed in PBS. Photomicrographs were made using the EVOS microscope described above.

Cell counts were performed using Adobe Photoshop. To determine cell densities, non-overlapping areas of 400 μm × 240 μm were defined in CA1 using the DAPI channel only, so that experimenter was blind to the density of IBA1^+^ or CD68^+^ cells during placement. A minimum of three sections were used to create a mean for each animal. Counts of objects touching the boundaries of the area of interests were only included from the north and east borders and excluded from the south and west borders. IBA1^+^ microglia were only counted if a DAPI positive nucleus was identified in the same focal plane. Microglia were only considered CD68^+^ if >30% of the soma had a signal for CD68.

*Preparing brain slices for electrophysiological recordings*

Patch clamp and field potential recordings were performed as described in [3] and [4]. Mice were decapitated, the brain rapidly removed and placed in ice-cold dissection artificial CSF (aCSF; containing (in mM): 125 NaCl, 2·4 KCl, 26 NaHCO_3_, 1·4 NaH_2_PO_4_, 20 D-glucose, 3 MgCl_2_, 0·5 CaCl_2_, pH 7·4, ~315 mOsm/l). A dorsal portion of the cortex was removed and the brain glued to a cutting stage using cyanoacrylate and submerged in slushed-ice dissection aCSF. Brain slices transverse to the long axis of the hippocampus were then cut at 400 μm using a vibrating microtome (Integraslice model 7550 MM, Campden Instruments, Loughborough, UK). Each slice was transferred immediately into a chamber containing ‘Carbogenated’ (95% O_2_/5% CO_2_; BOC Limited) dissection aCSF at room temperature (approximately 21°C) and after completion of sectioning, slices were then transferred into a chamber held at 36°C with the same dissection artificial cerebrospinal fluid. At 5-minute intervals, they were then consecutively transferred to physiological Ca^2+^ and Mg^2+^ ion concentrations (in mM): i) 1 Mg^2+^, 0·5 Ca^2+^; ii) 1 Mg^2+^, 1 Ca^2+^; iii) 1 Mg^2+^, 2 Ca^2+^.

*Patch-clamp recordings in brain slices*

Once allowed to recover for at least a further 40 minutes, a single slice was transferred to a submerged chamber and superfused (~2 ml/minute) with recording aCSF (containing (in mM): 125 NaCl, 2·4 KCl, 26 NaHCO_3_, 1·4 NaH_2_PO_4_, 20 D-glucose, 1 MgCl_2_, 2 CaCl_2_, bubbled with Carbogen). Individual CA1 pyramidal neurones were visualised using infrared-differential interference contrast microscopy on an upright microscope (Olympus BX50WI, 40× or 60× objective; Olympus UK). Patch electrodes (tip resistance 4–6 MΩ; model GC150F-7.5, 1·5 mm outer diameter × 0·86 mm inner diameter, Harvard Apparatus Ltd, Edenbridge, UK; or model 1B150F-3 1·5 mm outer diameter × 0·84 mm inner diameter, World Precision Instruments) were filled with a CsCl-based internal solution (containing (in mM): 140 CsCl, 5 HEPES, 10 EGTA, 2 Mg-ATP, pH7·4, ~290 mOsm/l) and whole cell voltage clamp recordings were then made according to standard methods [3]. Liquid junction potential, slow capacitance or series resistance were not compensated as errors introduced by these are calculated to be small when the membrane remains voltage-clamped at –70 mV. Fast capacitive transients, artifacts arising from the glass electrodes, were compensated once a GΩ seal was achieved. Patch-clamp recordings were performed using one of the following set ups: 1) EPC9/2 (HEKA Elektronik Dr Schulze GmbH, Lambrecht/Pfalz Germany) connected via the built-in ITC-16 digitiser board to a computer running Pulse software (version 8.80, HEKA); currents were amplified 10×, sequentially low-pass filtered at 10 kHz then 3 kHz and digitised at 10 kHz. 2) An Axopatch 1D (Molecular Devices, Sunnyvale, CA, USA) connected via a digitizer (Digidata 1322A, Molecular Devices; or 1401plus, Cambridge Electronic Design, Limited, Cambridge, UK) to a computer running WinWCP (for isolated events; Strathclyde University, UK) and WinEDR (for continuous recordings; Strathclyde University, UK). Responses were amplified 10×, low-pass filtered at 10 kHz (both via patch clamp amplifier) and subsequently at 3 kHz (Frequency Devices 902LPF or Brownlee Precision 440, USA); digitisation was set at 10 kHz.

For spontaneous and miniature currents, continuous recordings were made at a membrane holding potential of –70 mV. Spontaneous IPSCs were recorded in aCSF for 3-5 minutes. Subsequently, 6 μM gabazine (HelloBio, Bristol UK) was washed in for the remainder of the experiment. Spontaneous EPSCs were recorded for a period 15-30 minutes. Miniature EPSCs were isolated using 1 μM tetrodotoxin (Latoxan, France) and recorded at 30-60 minutes. Spontaneous and miniature currents were detected using the automated function within WinEDR and were required to remain over a threshold of 3 pA for 2 ms, with a dead time of 10 ms following an event. Currents identified by WinEDR were inspected by eye and only included if the rise time was <3 ms and faster than the decay. Inter-event interval was used to determine instantaneous frequencies (1/interval). WinWCP was used to measure amplitudes of individual identified synaptic currents and were determined as the magnitude from average baseline to the maximal negative deflection, taken as an average of 5 digitised points. To measure the decay, any complex currents were subsequently excluded and an average trace produced by aligning currents by their peak. A single exponential curve was fit from peak current to plateau of the decay (i.e. the current reached baseline) and the decay time constant τ (time to decay by 1 – e^–1^) determined from the fit.

Evoked currents were recorded in the presence of 6 µM gabazine by paired stimuli applied via a glass patch electrode filled with aCSF and positioned in the *stratum radiatum* of the CA1 ~150-300 μm from the recording electrode.

When unitary evoked currents were recorded to establish failure rates, minimal stimulation intensity was determined by applying ten repeat stimuli at incrementally increasing intensity from 1 V until a unitary-evoked current was observed then further increments until a current of double unitary amplitude was observed. Minimal stimulation was then set at the median voltage between unitary and double unitary current thresholds. At least 40 repeats at minimal stimulation were then applied at 10 s intervals to determine failure rates. Monosynaptic responses typically had ~4 ms latency between stimulus and synaptic current. Any responses with a latency >~8 ms were considered as a failure on the assumption that it was disynaptic in nature, rather than the monosynaptic response of interest.

For all other experiments, stimulus intensity (100 μs constant voltage pulse between 4 and 20 V, Digitimer DS2A-MkII or Grass SD9) was set at near-minimal stimulation, such that ~50-80% of stimuli successfully evoked an EPSC. At least 20 repeats at each inter-stimulus interval (25 and 50 ms) were then evoked. Any currents considered as disynaptic were excluded. Failures to evoke a synaptic current were included in the average. Responses were then averaged by aligning responses to the stimulus trigger. Amplitudes were determined from the baseline immediately prior to the given response. There were no significant differences between genotypes in the decay time constant (τ) of the evoked currents and therefore no differential interactions between first and second responses are expected. Paired-pulse ratios were calculated as the amplitude of the second response divided by the amplitude of the first response. As EPSCs were not fully recovered to baseline before the second stimulus at 25 ms interstimulus intervals were applied, the second responses were also measured from baseline of the first response. While this increased the paired-pulse ratio obtained, the qualitative outcome of the differences between genotypes was the same, irrespective of measurement method. Thus, the interpretation of the result would not change.

*Field potential recordings in brain slices*

Field potentials were recorded using standard protocols [4]. Slices were transferred as needed to a heated (30±1°C) submerged chamber, superfused with aCSF bubbled with Carbogen (~2 ml/minute) and allowed to recover for 1 h in the recording chamber. Recording and stimulating electrodes (filled with aCSF, resistance ~2 MΩ) were both positioned in *stratum radiatum* of the CA1 field, ~150-300 μm apart, to obtain a dendritic excitatory postsynaptic field potential (fEPSP) in the absence of GABA_A_ receptor antagonists. Field potentials were recorded via an AxoClamp 1B connected with a 1× gain headstage and amplified 10× via the 10Vm output. Recordings were controlled and recorded using WinWCP software, filtered at serially at 10 kHz (via Axoclamp) and 3 kHz (Frequency Devices 902LPF or Brownlee Precision 440, USA) and digitised at 10 kHz via a micro1401 interface (Cambridge Electrical Designs, UK) or Digidata 1332A (Molecular Devices). Stimulation (100 μs duration constant voltage, Digitimer DS2A- MkII or Grass SD9) was set at 30-50% of the intensity required to evoke a population spike or the maximum fEPSP amplitude obtained, whichever was the lower intensity. A stable baseline was recorded using pairs (50 ms interstimulus interval) of stimuli at 0.1 Hz for at least 15 minutes. LTP conditioning was applied at test-pulse stimulus intensity and consisted of 3 trains of tetani, each consisting of 20 pulses at 100 Hz, 1·5 s inter-train interval. Field EPSPs were then recorded under the baseline conditions for a further 1 h. Responses were averaged in blocks of 6 (i.e. 1 minute) and a linear fit made to the fEPSP from the initial steep slope to 50% of the peak, thus avoiding any contamination from either a population spike or inhibitory synaptic potentials, which are typically disynaptic when recorded with the electrode positioning used here and thus have a longer latency between stimulus and onset. For each slice, each 1-minute average fEPSP slope or paired-pulse ratio was expressed as percent of the respective average baseline. Magnitude of change was then summarised for that slice as the mean of the responses or ratios at 51-60 minutes post-conditioning.

*Hippocampal homogenisation and RNA extraction*

Whole hippocampus was extracted from the ipsilateral hemisphere that was drop-fixed in formalin for histological analyses and snap frozen in a microcentrifuge tube on dry ice and stored at -80ºC. The hippocampus was then homogenised using a Polytron PT 3000 at 7500 rpm in QIAzol RNA lysis reagent for 30 s. For phase separation, chloroform was added to the lysate followed by centrifugation at 12000g for 15 minutes. Total RNA was then extracted and DNA digested using an miRNeasy mini kit (Qiagen, USA) following the manufacturer’s instructions. The concentration and quality of the RNA produced were assessed using a NanoDrop 2000 spectrophotometer and the A260/A280 ratios calculated. Total RNA solutions were then snap-frozen on dry ice and stored at –80°C until use.

*Reverse transcription*

A 7·5 μl solution containing 2 μg of total RNA was created from the NanoDrop calculation of total RNA concentration for each sample. The samples were treated with RNaseOUT and amplification grade DNase I. This mixture was subsequently placed in a thermocycler (PTC-100 Programmable Thermal Controller; MJ Research, Inc.) for 15 minutes at 37°C for the digestion of contaminating DNA and deactivation of any RNase, followed by 15 minutes at 75°C for enzymatic denaturation. The reverse-transcription protocol was performed using a High-Capacity cDNA Reverse Transcription Kit with RNase Inhibitor (Applied Biosciences) according to the manufacturer’s instructions. Two negative controls were included and prepared in parallel reactions. The first lacked the reverse transcriptase enzyme and the second lacked RNA, both of which were substituted for nuclease-free water of the same volume. The thermocycle parameters were 10 minutes at 25°C followed by 2 hours at 37°C and finishing on 5 minutes at 85°C. The cDNA was then diluted in 60 μl of nuclease-free water to form 80 μl cDNA stock. The cDNA stock was then frozen and stored at –20°C.

*Real time-quantitative PCR*

cDNA samples were tested in triplicate in a 20 μl reaction volume in 96 well-plates. Each 20 μl reaction contained 10 μl SsoAdvanced Universal SYBR Green Supermix, 0·25 mM both forward and reverse primers, 7·5 μl nuclease-free water and 1·5 μl diluted cDNA neat solution. For *Actg1* detection*,* a 1:100 cDNA solution to nuclease-free water was used. For both *Trem2* and *Aif1* detection*,* a 1:10 cDNA solution to nuclease-free water was used. Both negative controls from the reverse-transcription protocol were included in the RT-qPCR to test for the presence of genomic DNA or contamination. Further, a third blank control was included that replaced the 1·5 μl cDNA solution for 1·5μl nuclease-free water in the 20 μl reaction.

Primers were designed by the authors and then produced by Eurofins Genomics (Germany) and tested to be specific:

*Actg1*:

Forward: TTTGAGCAAGAAATGGCTACTGC

Reverse: TATTGGCATACAGGTCTTTGCGG

*Aif1*:

Forward: GGAGACGTTCAGCTACTCTGAC

Reverse: CATCCACCTCCAATCAGGGC

*Trem2*:

Forward: GACCTCTCCACCAGTTTCTCC

Reverse: TCAGAGTGATGGTGACGGTTC

Plates were cycled in a CFX96 Real-Time System. The cycling parameters were: 95°C for 3 minutes followed by 40 cycles of [95°C for 30 s, 58°C for 30 s and 72°C for 30 s] the cycle finished at 72°C for 5minutes. A melt curve was produced by raising the temperature from 60°C to 90°C in 0·5°C increments every 5 s. All reactions were tested for a single peak, reflecting a single PCR product. The raw Ct values were averaged over their triplicates and results expressed as:

2^–(mean Ct value of gene of interest – mean Ct value of control gene)^.

*Microglial ablation*

Removal of microglia from the brain was achieved by feeding mice the colony stimulating factor 1 receptor (CSF1R) blocker, PLX5622 [Plexxikon Inc., CA, USA; 5]. At 1·5

(*App*^NL-G-F^) or 7 (*App*^NL-F^) months of age, shortly before the age when plaques were first detected, cages of male mice were randomly assigned to either control or test group. The standard grain-based diet mice are fed at the UCL Biological Services Unit (Envigo 2018 Teklad global 18% protein rodent diet) was gradually changed over a one week period to a refined diet (AIN-76A; Cat No D10001; Research Diets Inc, NJ, USA) supplemented with either vehicle, 300 or 1200 mg PLX5622 per kg diet. *App*^NL-G-F^ mice were fed for a further 2 months and *App*^NL-F^ mice for 3 months, before being killed for electrophysiological and histological analyses. Groups were staggard and interleaved to permit experiments to be performed on slices prepared from a single animal per day.

*Analyses*

All analyses were performed by an experimenter blinded to genotype. Where multiple cells/slices/sections were sampled from a single animal, a mean value was calculated for that animal, avoiding false replication from non-independent samples. Paired t-tests comparing baseline to post LTP induction were performed in GraphPad 8.2. ANOVA were performed in GraphPad Prism 8.2. When multiple comparable measures were made from a single animal (i.e. paired-pulse ratios at 25 and 50 ms inter-stimulus intervals), this measure was included as a repeated measure. Kruskal-Wallis non-parametric analyses were performed in GraphPad Prism 8.2 with each age-sex combination considered as a single independent variable (e.g. a comparison of males and females at 9, 14 and 18 months of age has 6 groups). Generalised linear mixed models (GLMM) were performed using SPSS 25 (IBM Software). The choice of post hoc correction was informed by the number of within family comparisons versus across family comparisons. Sigmoidal fits were made using GraphPad 8.2 with no weighting of Y values and the only constraint being that the bottom must be greater than zero. Comparisons of sigmoidal fits were assessed using an extra sums-of-squares F-test, with a null hypothesis ‘one curve for both data sets’; alternative hypothesis ‘different curves for each data set’.

**References**

1. Nystrom S, Psonka-Antonczyk KM, Ellingsen PG, Johansson LB, Reitan N, Handrick S, Prokop S, Heppner FL, Wegenast-Braun BM, Jucker M, et al: **Evidence for age-dependent in vivo conformational rearrangement within Abeta amyloid deposits.** *Acs Chem Biol* 2013, **8:**1128-1133.

2. Rasmussen J, Mahler J, Beschorner N, Kaeser SA, Hasler LM, Baumann F, Nystrom S, Portelius E, Blennow K, Lashley T, et al: **Amyloid polymorphisms constitute distinct clouds of conformational variants in different etiological subtypes of Alzheimer's disease.** *Proc Natl Acad Sci U S A* 2017, **114:**13018-13023.

3. Cummings DM, Liu W, Portelius E, Bayram S, Yasvoina M, Ho SH, Smits H, Ali SS, Steinberg R, Pegasiou CM, et al: **First effects of rising amyloid-beta in transgenic mouse brain: synaptic transmission and gene expression.** *Brain* 2015, **138:**1992-2004.

4. Medawar E, Benway TA, Liu W, Hanan TA, Haslehurst P, James OT, Yap K, Muessig L, Moroni F, Nahaboo Solim MA, et al: **Effects of rising amyloidbeta levels on hippocampal synaptic transmission, microglial response and cognition in APPSwe/PSEN1M146V transgenic mice.** *EBioMedicine* 2019, **39:**422-435.

5. Spangenberg E, Severson PL, Hohsfield LA, Crapser J, Zhang J, Burton EA, Zhang Y, Spevak W, Lin J, Phan NY, et al: **Sustained microglial depletion with CSF1R inhibitor impairs parenchymal plaque development in an Alzheimer's disease model.** *Nat Commun* 2019, **10:**3758.
