# Supplementary figures and images for: Knock-in models related to Alzheimer’s disease: synaptic transmission, plaques and the role of microglia
Source: Mol Neurodegener. 2021 Jul 15;16:47. doi: 10.1186/s13024-021-00457-0 (PMC8281661; doi:10.1186/s13024-021-00457-0)

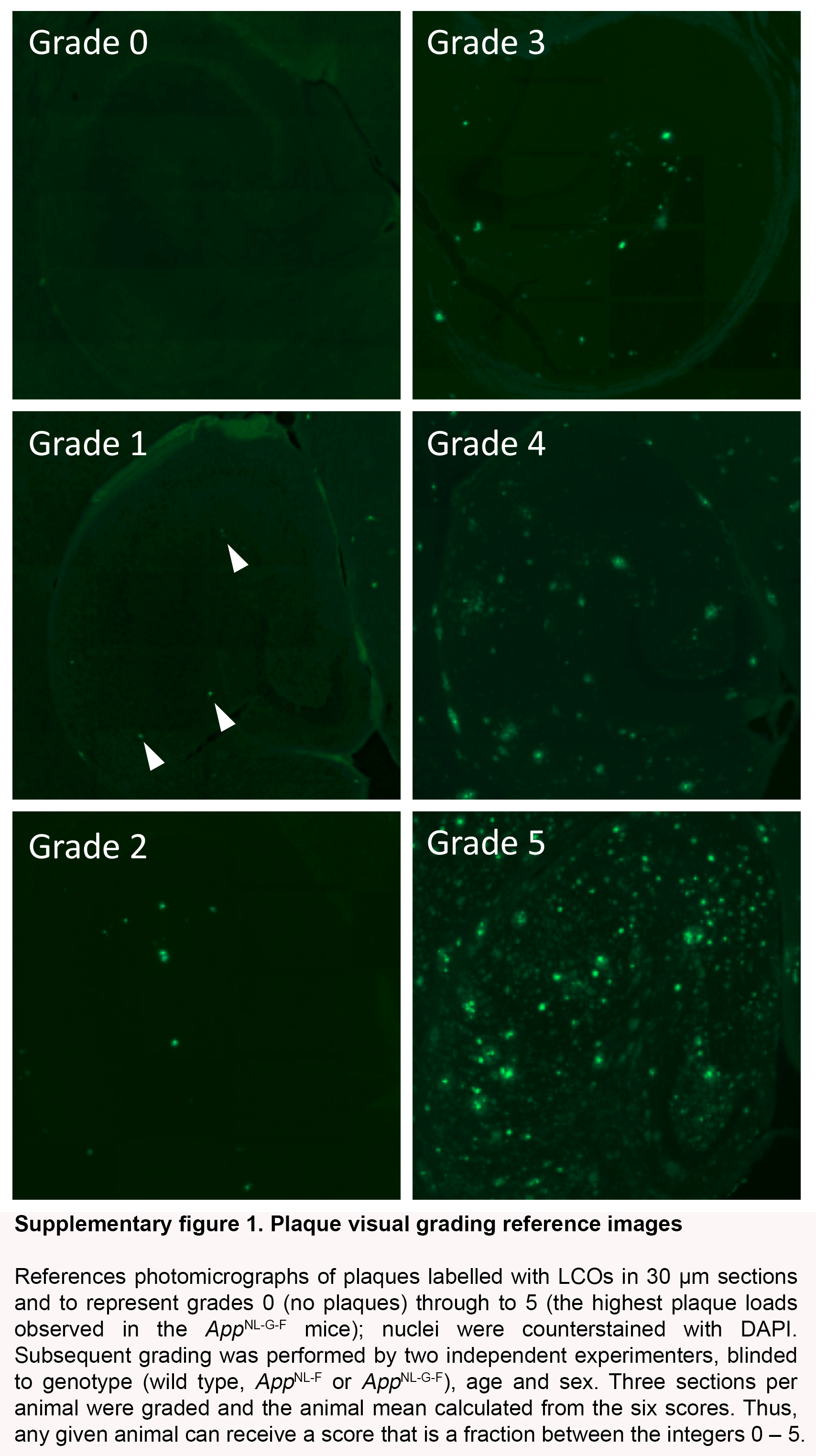

Supplement: Supplementary file 2 — Additional file 2.Supplementary figure 1. Plaque visual grading reference images. [file 13024_2021_457_MOESM2_ESM.tif]

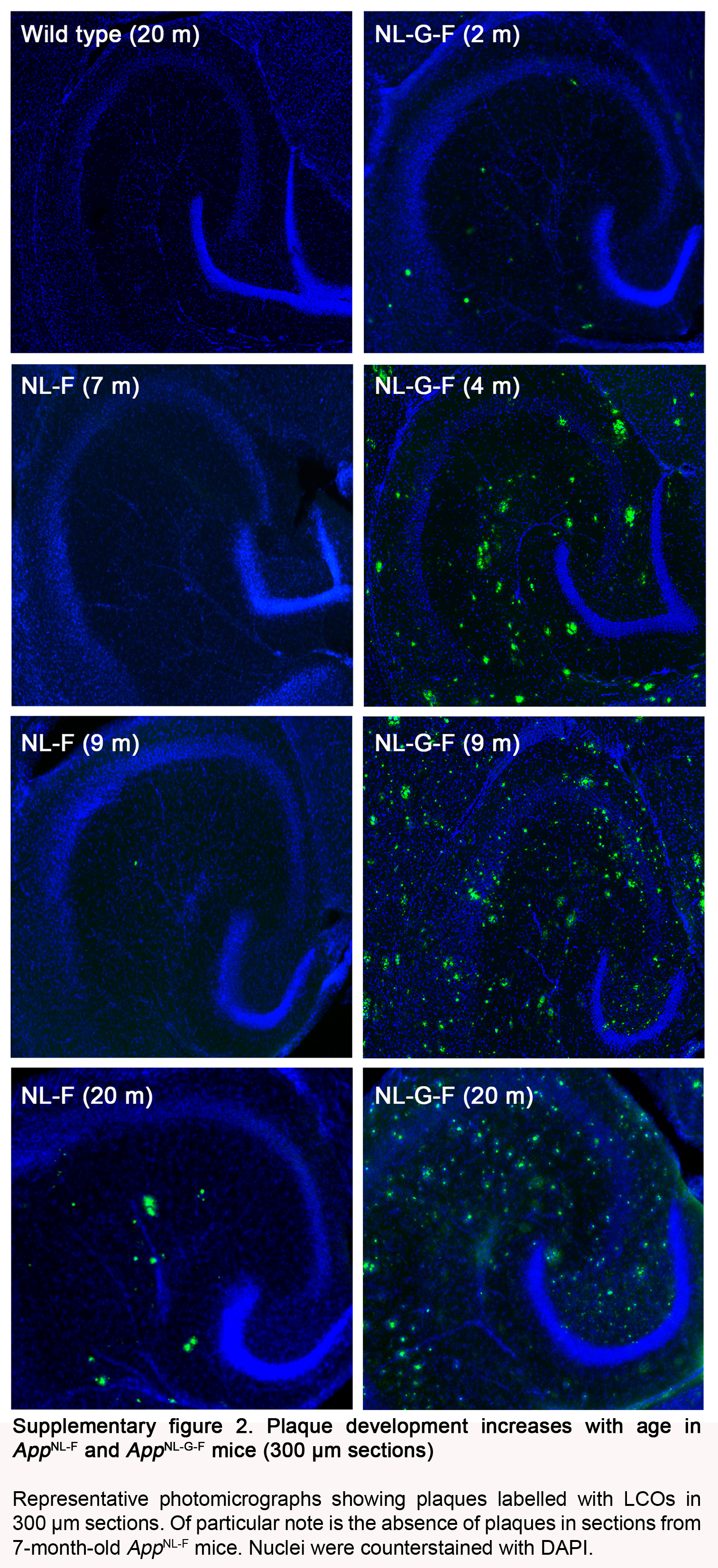

Supplement: Supplementary file 3 — Additional file 3.Supplementary figure 2. Plaque development increases with age in AppNL-F and AppNL-G-F mice (300 μm sections). [file 13024_2021_457_MOESM3_ESM.tif]

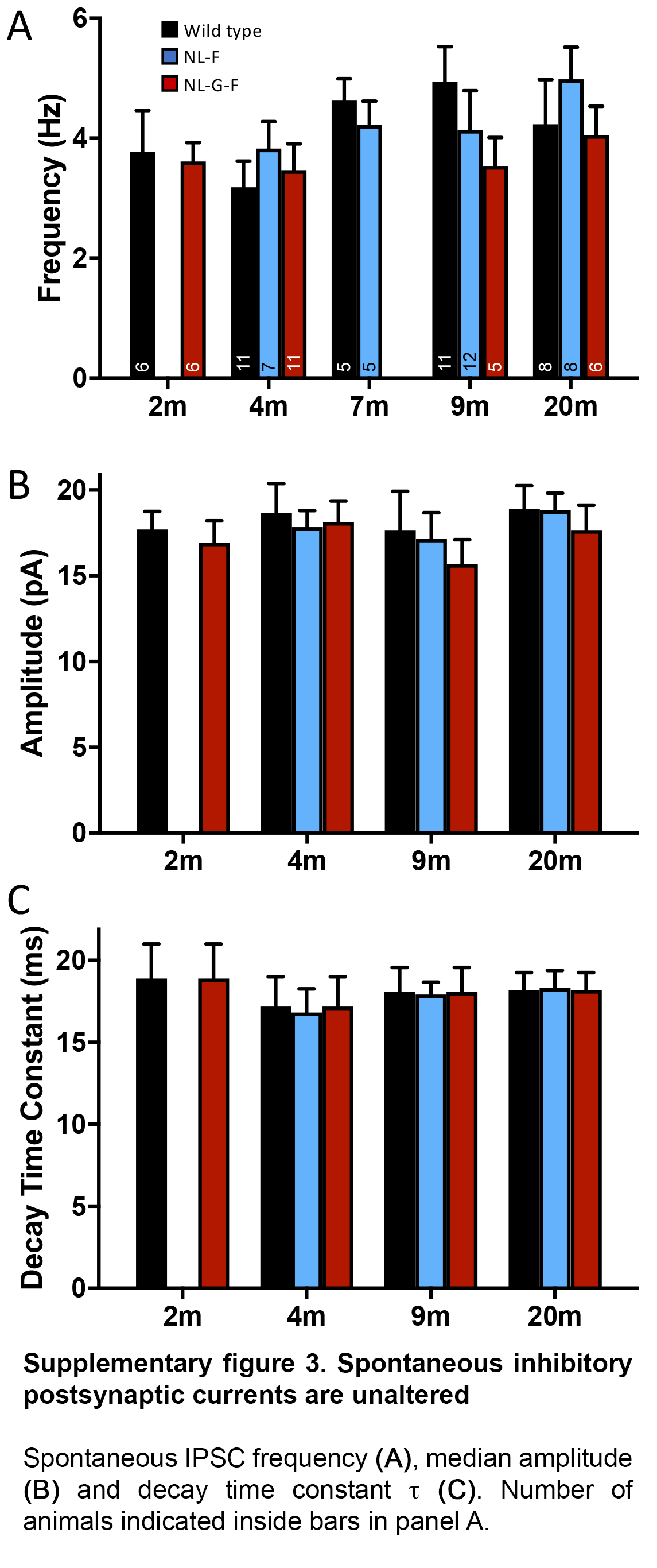

Supplement: Supplementary file 4 — Additional file 4.Supplementary figure 3. Spontaneous inhibitory postsynaptic currents are unaltered. [file 13024_2021_457_MOESM4_ESM.tif]

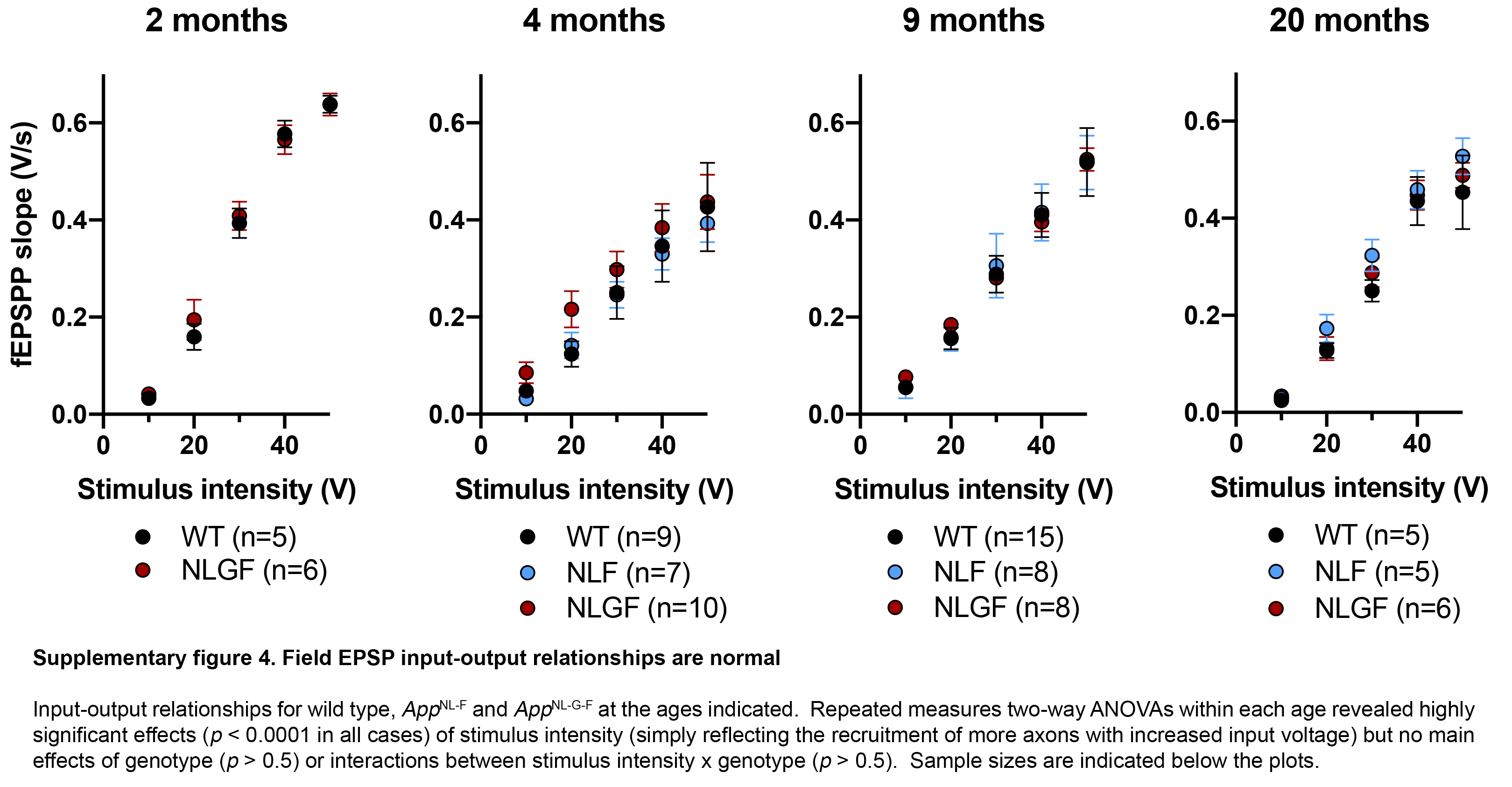

Supplement: Supplementary file 5 — Additional file 5.Supplementary figure 4. Field EPSP input-output relationships are normal. [file 13024_2021_457_MOESM5_ESM.tif]

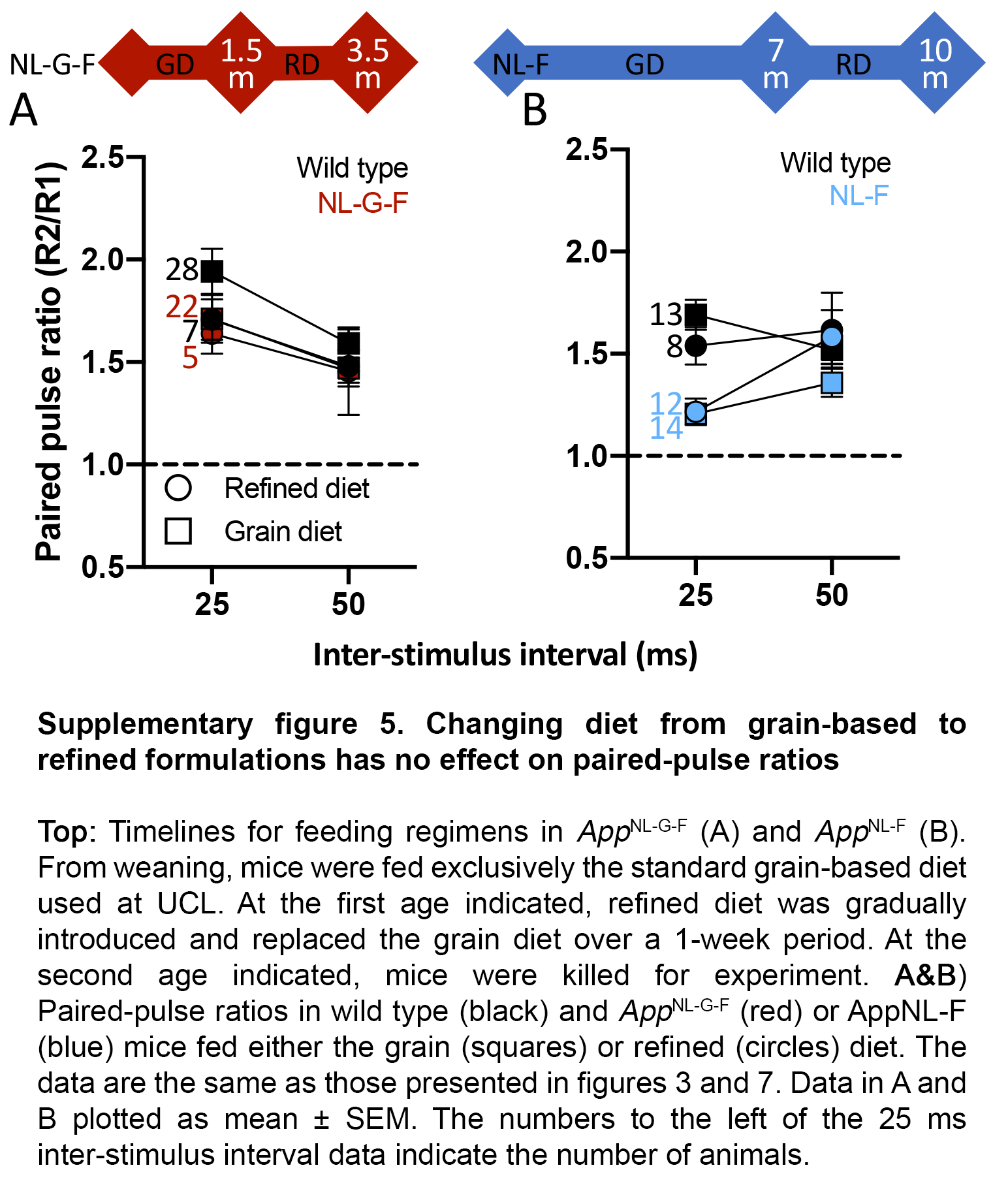

Supplement: Supplementary file 6 — Additional file 6.Supplementary figure 5. Changing diet from grain-based to refined formulations has no effect on paired-pulse ratios. [file 13024_2021_457_MOESM6_ESM.tif]
